# Intravascular Imaging in Patients With Complex Coronary Lesions and Chronic Kidney Disease

**DOI:** 10.1001/jamanetworkopen.2023.45554

**Published:** 2023-11-29

**Authors:** Woochan Kwon, Ki Hong Choi, Young Bin Song, Yong Hwan Park, Joo Myung Lee, Jong-Young Lee, Seung-Jae Lee, Sang Yeub Lee, Sang Min Kim, Kyeong Ho Yun, Jae Young Cho, Chan Joon Kim, Hyo-Suk Ahn, Chang-Wook Nam, Hyuck-Jun Yoon, Wang Soo Lee, Jin-Ok Jeong, Pil Sang Song, Joon-Hyung Doh, Sang-Ho Jo, Chang-Hwan Yoon, David Hong, Taek Kyu Park, Jeong Hoon Yang, Seung-Hyuk Choi, Hyeon-Cheol Gwon, Joo-Yong Hahn

**Affiliations:** 1 Samsung Medical Center, Sungkyunkwan University School of Medicine, Seoul, Korea; 2 Samsung Changwon Hospital, Sungkyunkwan University School of Medicine, Changwon, Korea; 3 Kangbuk Samsung Hospital, Sungkyunkwan University School of Medicine, Seoul, Korea; 4 Chungbuk National University Hospital, Chungbuk National University College of Medicine, Cheongju, Korea; 5 Chung-Ang University College of Medicine, Chung-Ang University Gwangmyeong Hospital, Gwangmyeong, Korea; 6 Wonkwang University Hospital, Iksan, Korea; 7 The Catholic University of Korea, Uijeongbu St Mary’s Hospital, Seoul, Korea; 8 Keimyung University Dongsan Hospital, Daegu, Korea; 9 Chung-Ang University College of Medicine, Chung-Ang University Hospital, Seoul, Korea; 10 Chungnam National University Hospital, Chungnam National University College of Medicine, Daejeon, Korea; 11 Inje University Ilsan Paik Hospital, Goyang, Korea; 12 Cardiovascular Center, Hallym University Sacred Heart Hospital, Anyang, Korea; 13 Seoul National University Bundang Hospital, Seongnam-si, Gyeonggi-do, Korea

## Abstract

**Importance:**

As patients with chronic kidney disease (CKD) are more likely to have complex coronary lesions, intravascular imaging guidance in percutaneous coronary intervention (PCI) for this population could be potentially beneficial.

**Objectives:**

To investigate whether the outcomes of intravascular imaging–guided procedural optimization would be different according to the presence of CKD.

**Design, Setting, and Participants:**

This was a prespecified substudy of RENOVATE-COMPLEX-PCI, a recently published multicenter randomized clinical trial in Korea studying the benefits of intravascular imaging for complex coronary lesions. Patients with complex coronary lesions, with or without CKD, were enrolled between May 2018 and May 2021. Data were analyzed from January to June 2023.

**Interventions:**

PCI in each group was done either under the guidance of intravascular imaging or angiography alone.

**Main Outcomes and Measures:**

The primary end point was target vessel failure (TVF) at the 3-year point, defined as a composite of cardiac death, target vessel–related myocardial infarction, or clinically driven target vessel revascularization.

**Results:**

A total of 1639 patients (1300 male [79.3%]) treated with PCI for complex coronary lesions were stratified into CKD (296 participants) and non-CKD (1343 participants) groups. The mean (SD) age of each group was 70.3 (9.4) and 64.5 (10.1) years, and mean (SD) estimated serum creatinine was 2.9 (5.3) and 0.8 (0.2) mg/dL for CKD and non-CKD groups, respectively. Intravascular imaging–guided revascularization was associated with significantly lower incidence of the primary end point compared with angiography-guided revascularization in both CKD (13.3% vs 23.3%; hazard ratio [HR], 0.51; 95% CI, 0.27-0.93; *P* = .03) and non-CKD (6.4% vs 9.9%; HR, 0.66; 95% CI, 0.44-0.99; *P* = .05) groups. The significantly lower incidence of the primary end point was mainly associated with the lower risk of cardiac death or target vessel–related myocardial infarction (9.4% vs 22.2%; HR, 0.39; 95% CI, 0.20-0.76; *P* = .006) in the CKD group and by target vessel revascularization (3.0% vs 5.5%; HR, 0.55; 95% CI, 0.30-0.99; *P* = .05) in the non-CKD group. Those with a glomerular filtration rate of at least 30 mL/min/1.73m^2^ and less than 60 ml/kg/1.73m^2^ showed the greatest benefit from imaging-guided complex PCI (8.8% vs 21.2%; HR, 0.28; 95% CI, 0.11-0.68; *P* = .02).

**Conclusions and Relevance:**

In this prespecified cohort substudy of the Randomized Controlled Trial of Intravascular Imaging Guidance versus Angiography-Guidance on Clinical Outcomes After Complex Percutaneous Coronary Intervention trial, intravascular imaging guidance showed clinical benefit over angiography guidance in reducing the risk of TVF, regardless of the presence of CKD. The greatest benefits of imaging-guided complex PCI were observed in stage 3 CKD.

**Trial Registration:**

ClinicalTrials.gov Identifier: NCT03381872

## Key Points

**Question:**

Is intravascular imaging beneficial in patients with complex coronary lesions and chronic kidney disease?

**Findings:**

In this prespecified cohort substudy of the RENOVATE-COMPLEX-PCI trial with 1639 patients with or without chronic kidney disease, intravascular imaging–guided revascularization was associated with significantly lower incidence of the target vessel failure (a composite of cardiac death, myocardial infarction, or target vessel revascularization) compared with angiography-guided revascularization, whether with chronic kidney disease or not.

**Meaning:**

These findings suggest that in patients with complex coronary artery lesions, intravascular imaging–guided revascularization was superior to angiography-guided revascularization in reducing the risk of target vessel failure, regardless of kidney function.

## Teaser

This cohort study evaluates whether the outcomes of intravascular imaging–guided procedural optimization would be different according to the presence of chronic kidney disease.

## Introduction

Chronic kidney disease (CKD) is a prevalent condition associated with increased cardiovascular morbidity and mortality.^1^ Patients with CKD often present with complex coronary lesions, posing significant challenges for a percutaneous coronary intervention (PCI) procedure.^2^ In addition, coronary lesions in patients with CKD are typically longer, have a higher plaque burden,^3^ and are more commonly accompanied by calcification,^4^ all of which are classified as complex characteristics.

Intravascular imaging tools, such as intravascular ultrasound (IVUS) and optical coherence tomography (OCT), are useful aids in optimizing stent implantation and securing positive clinical outcomes.^5^ Benefits of IVUS for complex coronary lesions have been demonstrated in several previous studies including the recently published Randomized Controlled Trial of Intravascular Imaging Guidance versus Angiography-Guidance on Clinical Outcomes After Complex Percutaneous Coronary Intervention (RENOVATE-COMPLEX PCI).^6,7,8,9,10,11^ Although there is a growing body of evidence supporting the use of intravascular imaging–guided PCI in patients with coronary artery disease, there are limited data focusing on patients with CKD and complex coronary lesions. As patients with CKD are more likely to be complicated with complex coronary lesions, it can be assumed that these benefits could be translated to or even amplified in patients with CKD. Understanding the impact of intravascular imaging–guided PCI in this high-risk population is crucial for improving patient outcomes and refining treatment strategies. We performed a prespecified subgroup analysis of the RENOVATE-COMPLEX-PCI aimed at investigating whether the benefit of IVUS or OCT would be maintained in the CKD population among patients undergoing complex PCI.

## Methods

### Trial Design and Patient Selection

The RENOVATE-COMPLEX-PCI was an investigator-initiated, randomized, open-label, multicenter, superiority trial at 20 sites in Korea. The design and primary results have been described previously.^11^ In brief, patients aged 19 years or older undergoing PCI for complex coronary artery lesions were enrolled. Complex coronary artery lesions were defined as true bifurcation lesions with side branches 2.5 mm or greater, chronic total occlusion, unprotected left main disease, long coronary lesions, multivessel PCI, multiple stents needed, in-stent restenosis, severely calcified lesions, or coronary ostial lesions. The trial protocol was approved by the institutional review board at each participating site. Written informed consent was obtained from all patients before randomization. All participating centers, trial personnel, and detailed inclusion and exclusion criteria of this trial are listed in the eAppendix in Supplement 1. From May 2018 to May 2021, patients enrolled in the trial were stratified according to the presence of CKD for the current prespecified substudy. We followed the Strengthening the Reporting of Observational Studies in Epidemiology (STROBE) reporting guideline.

### Randomization and Treatment

Eligible patients with amenable complex coronary artery lesions were randomized in a 2:1 ratio to the intravascular imaging–guided PCI group or the angiography-guided PCI group. Randomization was performed by a web-based program run by an independent organization and stratified according to clinical presentation and participating sites.

Standard PCI was performed according to the current clinical guidelines.^12,13^ Detailed protocols for PCI technique, intravascular imaging use, image acquisition, optimization criteria of the stented segment, and medical treatment after PCI are described in the eAppendix in Supplement 1. The choice of intravascular imaging modalities was left to the operator’s discretion. The timing of intravascular imaging was not restricted during index procedures, but intravascular imaging evaluation for optimization of the stented segment after PCI was mandatory. The optimal cutoff for stent expansion was an absolute minimum stent area of more than 5.5 mm^2^ as determined by IVUS or more than 4.5 mm^2^ as determined by OCT in nonleft main stenosis. For left main lesions, the cutoff values for optimization were an absolute minimum stent area more than 7 mm^2^ for distal left main and more than 8 mm^2^ for proximal left main.^14^ Regardless of assigned group, current clinical guideline-directed medical therapy was conducted.^12,13^

### Definitions and End Points

CKD was defined if the patient previously received a diagnosis of CKD (supported either by medical record or history-taking) or if the estimated glomerular filtration rate (GFR) was below 60mL/min/1.73m^2^.^15^ The primary end point was target vessel failure (TVF), which is a composite of cardiac death, target vessel–related myocardial infarction (MI), or clinically driven target vessel revascularization. Secondary end points included individual components of the primary end point, TVF without procedure-related MI, a composite of cardiac death or target vessel–related MI, definite stent thrombosis, and incidence of contrast-induced nephropathy (CIN). The definition of spontaneous MI was from the third universal definition of MI.^16^ The definition of procedure-related MI was based on the Society for Cardiovascular Angiography and Interventions.^17^ CIN is defined as an increase in serum creatinine of 0.5 mg/dL or greater (to convert to millimoles per liter, multiply by 88.4) or 25% or greater from baseline within 48 to 72 hours after contrast agent exposure.

Patient follow-up was conducted at 1, 6, and 12 months and annually thereafter. The clinical follow-up was completed in May 2022. Patients missing the scheduled follow-up visits were contacted by telephone. For patients lost to follow-up, mortality status was confirmed using the Korean National Health Insurance database.

### Statistical Analysis

The full statistical analysis plan and sample size calculation of the RENOVATE-COMPLEX-PCI were previously described in detail.^11^ Detailed analyses are provided in the eMethods in Supplement 1. No imputation methods were used to infer missing values of baseline variables. Cumulative incidence of end points was evaluated by Kaplan-Meier analyses and significance level was assessed with the log-rank test. Treatment effects were estimated by Cox proportional hazard regression models with hazard ratio (HR) and 95% CI. In multivariable analysis, the covariates that were clinically relevant were included. Multivariable models for Cox regression to compare outcomes between the imaging-guided PCI and the angiography-guided PCI included age, sex, acute coronary syndrome, history of PCI, 3 or more complex coronary lesions, use of adjunctive noncompliant balloon, and dialysis (only in CKD group) as covariables. Restricted cubic spline curves with 3 knots were used to evaluate the continuous effects of GFR on the primary end point. All probability values were 2-sided, and *P* values less than .05 were considered statistically significant. Statistical analyses were performed using R version 4.1.2 (R Project for Statistical Computing). Data were analyzed from January to June 2023.

## Results

### Baseline Demographics

Of the 1639 patients (1300 male [79.3%]) enrolled in this trial, 296 patients (mean [SD] age, 70.3 [9.4] years) had CKD (203 patients undergoing imaging-guided PCI and 93 patients undergoing angiography-guided PCI) and 1343 patients (mean [SD] age, 64.5 [10.1] years) did not have CKD (889 patients undergoing imaging-guided PCI and 454 patients undergoing angiography-guided PCI) (eFigure 1 in Supplement 1). Among patients with CKD assigned to the intravascular imaging–guided PCI, IVUS was used in 84.2% (171 patients), while OCT was used in only 14.3% (29 patients). The use of OCT in the CKD population was significantly less than in patients without CKD (eFigure 2 in Supplement 1). The distribution of GFR is shown in eFigure 3 in Supplement 1, and median (IQR) creatinine in the CKD and non-CKD groups was 1.4 (1.2-2.0) and 0.8 (0.7-1.0) mg/dL, respectively. Among the population with CKD, 52 patients (17.6%) were on dialysis before enrollment. eTable 1 in Supplement 1 presents differences in baseline characteristics between the CKD and non-CKD groups. Compared with patients without CKD, those with CKD were older; were less likely to be male; and were more likely to have cardiovascular risk factors, including hypertension, diabetes, history of PCI, MI, stroke, and peripheral vascular disease. Left ventricular ejection fraction and the proportion of patients that presented with acute coronary syndrome were significantly lower in the CKD group than in the non-CKD group.

Baseline characteristics between the imaging-guided and the angiography-guided PCI groups stratified by the presence of CKD are shown in Table 1. Baseline demographic characteristics in the 2 groups of the CKD population were balanced, except for the proportion of dialysis before enrollment. In the non-CKD group, there were no significant differences in baseline demographic characteristics between the 2 allocated groups.

**Table 1.** Baseline Characteristics Stratified by Presence of Chronic Kidney Disease (CKD) and Allocation Group

Demographics	Participants, No. (%)			
	CKD (n = 296)		Non-CKD (n = 1343)	
	Intravascular imaging–guided (n = 203)	Angiography-guided (n = 93)	Intravascular imaging–guided (n = 889)	Angiography-guided (n = 454)
Age, mean (SD), y	70.3 (9.6)	70.6 (8.9)	64.2 (10.1)	65.1 (10.0)
Sex				
Male	151 (74.4)	68 (73.1)	718 (80.8)	363 (80.0)
Female	52 (25.6)	25 (26.9)	171 (19.2)	91 (20.0)
Initial presentation				
Stable ischemic heart disease	104 (51.2)	51 (54.8)	71 (8.0)	45 (9.9)
Acute coronary syndrome	99 (48.8)	42 (45.2)	357 (40.2)	179 (39.4)
Medical history				
Hypertension	166 (81.8)	66 (71.0)	516 (58.0)	257 (56.6)
Diabetes	98 (48.3)	47 (50.5)	296 (33.3)	176 (38.8)
Dyslipidemia	107 (52.7)	38 (40.9)	453 (51.0)	242 (53.3)
Current smoking	35 (17.2)	11 (11.8)	177 (19.9)	84 (18.5)
Previous PCI	67 (33.0)	26 (28.0)	201 (22.6)	101 (22.2)
Previous myocardial infarction	20 (9.9)	13 (14.0)	55 (6.2)	29 (6.4)
Previous stroke	22 (10.8)	7 (7.5)	48 (5.4)	35 (7.7)
Peripheral vascular disease	8 (3.9)	9 (9.7)	19 (2.1)	8 (1.8)
LV ejection fraction, %^a^	52.1 (14.6)	54.7 (12.3)	59.9 (10.6)	60.3 (10.5)
Creatinine, mean (SD), mg/dL	2.7 (5.9)	3.1 (3.8)	0.8 (0.2)	0.9 (0.2)
Creatinine, median (IQR), mg/dL	1.4 (1.2-1.9)	1.5 (1.2-3.4)	0.8 (0.7-1.0)	0.8 (0.7-1.0)
Estimated GFR, mL/min/1.73 m^2^	42.9 (18.5)	38.7 (20.9)	90.2 (20.3)	88.7 (21.3)
Dialysis	27 (13.3)	25 (26.9)	0	0
Target lesion characteristics				
Complex coronary lesions				
True bifurcation (Medina 1,1,1; 1,0,1; or 0,1,1)	39 (19.2)	16 (17.2)	194 (21.8)	110 (24.2)
Chronic total occlusion (≥3 mo of occlusion)	44 (21.7)	14 (15.1)	176 (19.8)	85 (18.7)
Unprotected left main disease	25 (12.3)	13 (14.0)	113 (12.7)	41 (9.0)
Long coronary lesion (stent length ≥38 mm)	118 (58.1)	54 (58.1)	499 (56.1)	227 (50.0)
Multivessel PCI (≥2 major coronary arteries treated)	74 (36.5)	40 (43.0)	335 (37.7)	173 (38.1)
Multiple stents implanted (≥3 more stents)	44 (21.7)	16 (17.2)	164 (18.4)	81 (17.8)
In-stent restenosis lesion	44 (21.7)	15 (16.1)	114 (12.8)	63 (13.9)
Severely calcified lesion	29 (14.3)	19 (20.4)	128 (14.4)	55 (12.1)
Ostial coronary lesion	31 (15.3)	14 (15.1)	151 (17.0)	55 (12.1)
No. of complex coronary lesions ≥3	68 (33.5)	30 (32.3)	284 (31.9)	123 (27.1)
Arteries with stenosis				
1 Vessel disease	44 (21.7)	26 (28.0)	298 (33.5)	158 (34.8)
2 Vessel disease	91 (44.8)	30 (32.3)	329 (37.0)	171 (37.7)
3 Vessel disease	68 (33.5)	37 (39.8)	262 (29.5)	125 (27.5)
Procedural characteristics				
Total No. of target lesions treated, mean (SD)	1.5 (0.8)	1.6 (0.8)	1.5 (0.7)	1.5 (0.7)
Radial access	122 (60.1)	52 (55.9)	705 (79.3)	374 (82.4)
Intravascular imaging devices used				
Intravascular ultrasound	171 (84.2)	3 (3.2)	634 (71.3)	9 (2.0)
Optical coherence tomography	29 (14.3)	0	250 (28.1)	0
Not done	3 (1.5)	90 (96.8)	5 (0.6)	445 (98.0)
Adjunctive noncompliant balloon used	132 (65.0)	53 (57.0)	670 (75.4)	279 (61.5)
Rotablator used	8 (3.9)	2 (2.2)	29 (3.3)	14 (3.1)
Treatment devices used				
Drug-eluting stent	193 (95.1)	90 (96.8)	871 (98.0)	440 (96.9)
Drug-coated balloon angioplasty	10 (4.9)	3 (3.2)	18 (2.0)	14 (3.1)
Total No. of devices used per patient, mean (SD)	2.0 (1.2)	2.1 (1.1)	1.9 (1.0)	1.8 (1.0)
Dimensions of devices, mean (SD), mm				
Mean diameter	3.1 (0.4)	3.1 (0.4)	3.2 (1.5)	3.1 (0.4)
Total length	57.3 (37.0)	60.1 (32.0)	55.6 (32.0)	53.6 (31.7)
Volume of contrast media used, , mean (SD), ml	198.9 (115.6)	185.6 (125.5)	217.6 (118.9)	195.4 (108.3)
Procedural time, median (IQR), min	73.0 (50.0-100.0)	60.0 (42.5-75.5)	69.5 (51.0-94.0)	52.0 (40.0-74.0)
Procedural success	200 (98.5)	91 (97.8)	885 (99.6)	452 (99.6)

Abbreviations: GFR, glomerular filtration rate; LV, left ventricle; PCI, percutaneous coronary intervention.

SI conversion factor: To convert creatinine to micromoles per liter, multiply by 76.25.

^a^ Of the total 1639 patients, 104 (6.3%) had no LV ejection fraction.

### Angiographic and Procedural Characteristics

A comparison of angiographic and procedural characteristics between CKD and non-CKD groups is shown in eTable 2 in Supplement 1. The CKD group had more multivessel disease and in-stent restenosis lesions compared with the non-CKD group. For procedural characteristics, patients with CKD were less likely to receive transradial intervention, adjunctive noncompliant balloon inflation, drug-eluting stent implantation, successful revascularization, and successful imaging-guided optimization than those without CKD (eTable 2 in Supplement 1). The amount of contrast used during PCI was significantly less in the CKD group than in the non-CKD group.

Angiographic characteristics and lesion complexity between the 2 randomly assigned strategies were generally well balanced, regardless of the presence of CKD (Table 1). In both the CKD and non-CKD groups, procedural time was longer in patients assigned to imaging-guided PCI than in those assigned to angiography-guided PCI. In the non-CKD group, a larger amount of contrast was used in patients who received imaging-guided PCI than in those who received angiography-guided PCI, but there was no significant difference in the amount of contrast between the 2 treatment strategies in the CKD group (Table 1).

In lesion-level analysis, the location of the target vessel was well balanced between patients with or without CKD. Quantitative coronary angiography findings are presented in eTable 3 in Supplement 1. No significant differences were observed in patients with CKD regarding the pre-PCI and post-PCI quantitative coronary angiography data between the 2 groups. In patients without CKD, pre-PCI proximal reference diameter and post-PCI minimum lumen diameter were significantly larger in the imaging-guided PCI group than in the angiography-guided PCI group. In the imaging-guided PCI group, patients with CKD were less likely to achieve an optimal stent expansion and showed a lower stent expansion index compared with those without CKD (eTable 4 in Supplement 1).

### End Points

**Figure 1.**
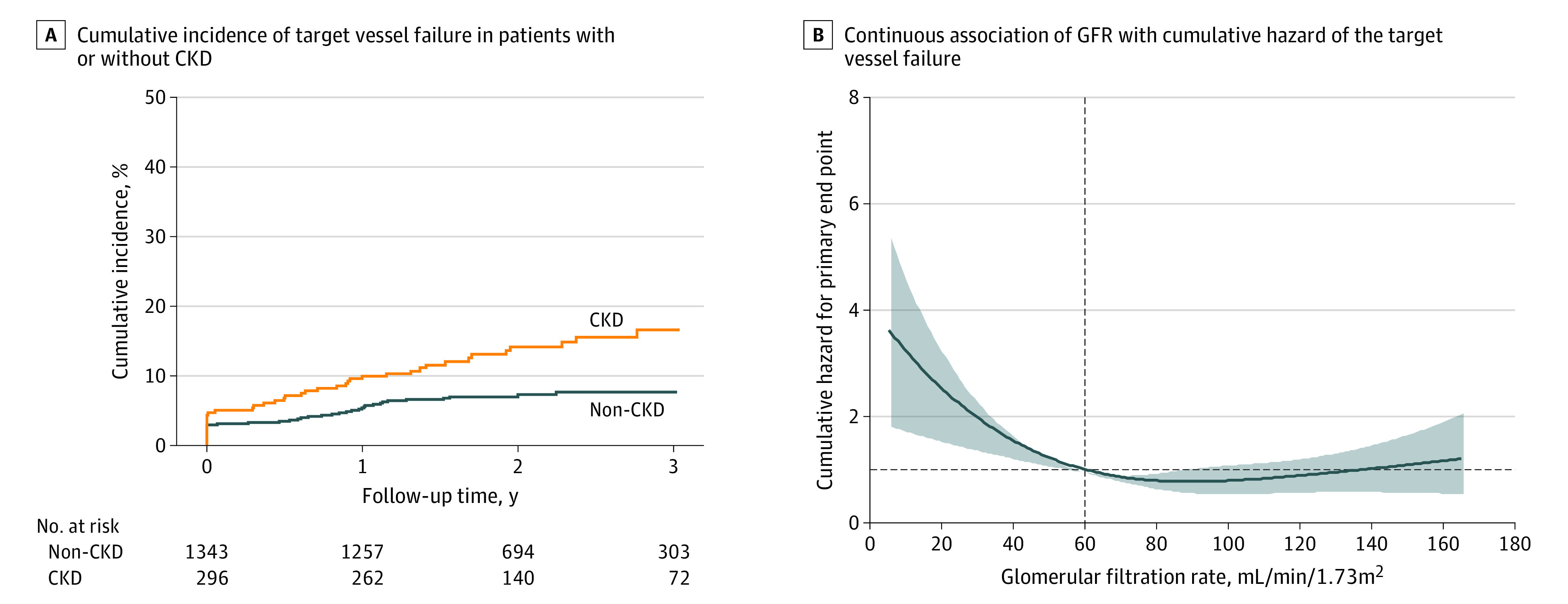
Comparison of Primary End Points According to Chronic Kidney Disease (CKD) and Glomerular Filtration Rate (GFR) A, The Kaplan-Meier curve shows the cumulative incidence of target vessel failure in patients with (orange line) or without (blue line) CKD who underwent complex percutaneous coronary intervention. B, Continuous association of GFR with cumulative hazard of the target vessel failure is presented. Adjusted variables included age over 70 years, sex, acute coronary syndrome, hypertension, diabetes, dyslipidemia, history of percutaneous coronary intervention, left ventricular systolic ejection fraction below 40%, and number of diseased vessels.

Figure 1 shows the association between baseline kidney function and primary end point. Patients with CKD had a 2-fold higher risk for the primary end point than those without CKD (16.5% vs 7.6%; HR, 2.03; 95% CI, 1.41-2.92; *P* < .001) (Figure 1A). In addition, there was a significant reverse association between baseline GFR value and the risk of primary end point (HR [per 10 mL/min/1.73 m^2^ GFR decrease], 1.10; 95% CI, 1.03-1.17; *P* = .002) (Figure 1B). These results were consistent even after conducting multivariable analyses.

**Figure 2.**
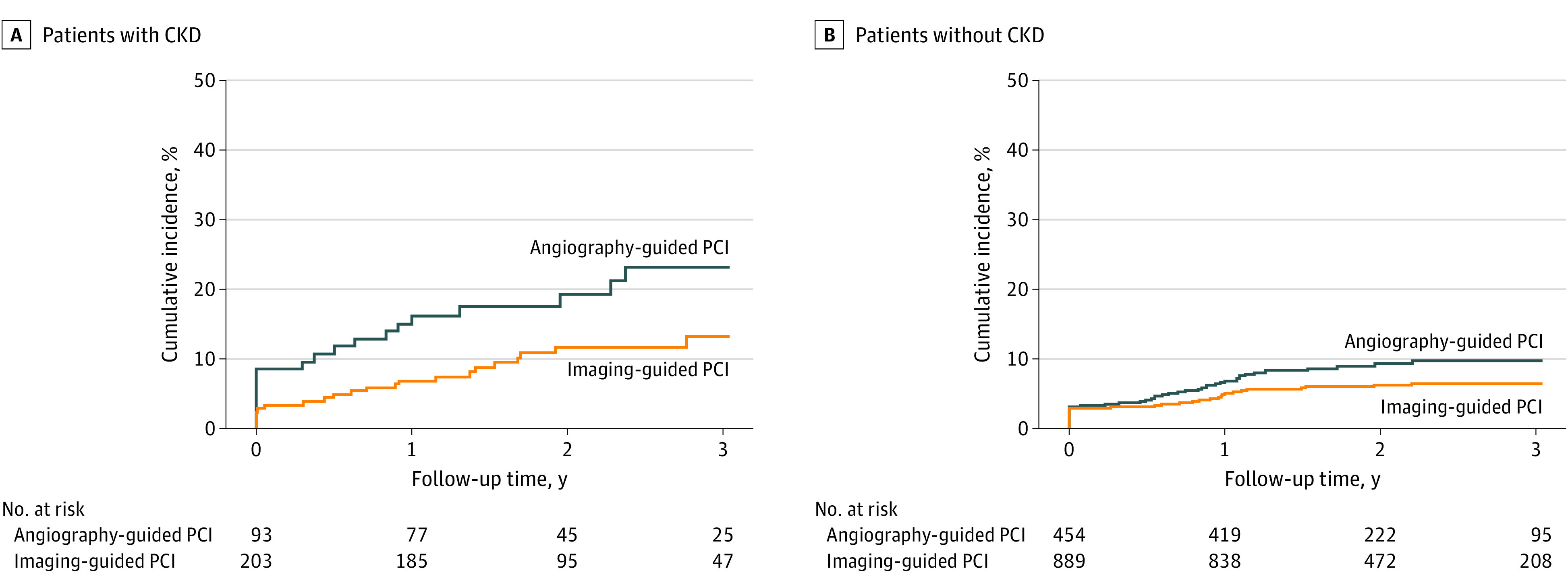
Comparison of Target Vessel Failure Between Imaging-Guided and Angiography-Guided Percutaneous Coronary Intervention (PCI), Stratified by the Presence of Chronic Kidney Disease (CKD) The Kaplan-Meier curve shows the cumulative incidence of target vessel failure in intravascular imaging-guided PCI (orange line) and angiography-guided PCI (blue line) for patients with (A) or without (B) CKD.

Among the CKD population, the primary end point occurred in 22 of 203 patients in the intravascular imaging–guided group and 19 of 93 patients in the angiography-guided group (13.3% vs 23.3%; HR, 0.51; 95% CI, 0.27-0.93; *P* = .03) (Table 2 and Figure 2A). The significantly lower risk of TVF in the intravascular imaging–guided group was associated with the lower incidence of cardiac death or target vessel–related MI (9.4% vs 22.2%; HR, 0.39; 95% CI, 0.20-0.76; *P* = .006) (eFigure 4 in Supplement 1). Similar results were observed even after adjustment for confounding variables including dialysis (Table 2). Although there was a numerically lower risk of CIN in the intravascular imaging–guided PCI group, the observed difference was not statistically significant (Table 2).

**Table 2.** Primary and Secondary End Points in Patients With Chronic Kidney Disease

End point	Participants, No. (%)^a^			HR (95% CI)	
	Total (N = 296)	Intravascular imaging–guided (n = 203)	Angiography-guided (n = 93)	Univariable analysis	Multivariable analysis^b^
Primary end point					
Target vessel failure	41 (16.5)	22 (13.3)	19 (23.3)	0.51 (0.27-0.93)	0.53 (0.28-0.99)
Secondary end point					
Target vessel failure without procedure-related MI	30 (12.9)	18 (11.3)	12 (15.2)	0.68 (0.33-1.42)	0.74 (0.35-1.60)
Cardiac death or target vessel–related MI	34 (13.5)	16 (9.4)	18 (22.2)	0.39 (0.20-0.76)	0.41 (0.21-0.83)
All-cause death	34 (15.4)	22 (15.5)	12 (15.4)	0.84 (0.42-1.70)	0.94 (0.45-1.97)
Cardiac death	18 (7.8)	9 (5.8)	9 (12.0)	0.46 (0.18-1.17)	0.51 (0.19-1.34)
MI					
Any	21 (8.6)	10 (6.9)	11 (12.8)	0.40 (0.17-0.95)	0.45 (0.19-1.09)
Target vessel–related MI	19 (6.9)	8 (4.2)	11 (12.8)	0.32 (0.13-0.79)	0.34 (0.13-0.87)
Spontaneous MI	7 (2.9)	3 (5.4)	4 (1.8)	0.34 (0.08-1.51)	0.45 (0.09-2.23)
Procedure-related MI	13 (4.4)	5 (2.5)	8 (8.6)	0.28 (0.09-0.85)	0.29 (0.09-0.91)
Nontarget vessel–related MI	2 (1.7)	2 (2.7)	0	NA	NA
Repeat revascularization					
Any	18 (8.5)	14 (10.0)	4 (5.5)	1.60 (0.53-4.86)	1.78 (0.55-5.74)
Target vessel revascularization	13 (5.7)	9 (5.7)	4 (5.5)	0.99 (0.31-3.22)	0.99 (0.29-3.37)
Target lesion revascularization	9 (3.7)	7 (4.3)	2 (2.3)	1.55 (0.32-7.46)	1.65 (0.32-8.59)
Nontarget vessel revascularization	7 (3.9)	7 (5.8)	0	NA	NA
Definite stent thrombosis	3 (1.1)	1 (0.5)	2 (2.2)	0.23 (0.02-2.51)	0.27 (0.02-3.11)
Contrast-induced nephropathy	16 (5.4)	9 (4.4)	7 (7.5)	0.60 (0.22-1.62)	0.56 (0.19-1.67)

Abbreviations: HR, hazard ratio; MI, myocardial infarction; NA, not applicable.

^a^ Percentages are 3-year Kaplan-Meier estimates.

^b^ Adjusted variables for multivariable analysis were age, sex, acute coronary syndrome, history of percutaneous coronary intervention, 3 or more complex coronary lesions, use of adjunctive noncompliant balloon, and dialysis.

Among the non-CKD population, the primary end point occurred in 54 of 889 patients in the intravascular imaging–guided PCI group and 41 of 454 patients in the angiography-guided PCI group (6.4% vs 9.9%; HR, 0.66; 95% CI, 0.44-0.99; *P* = .048) (Table 3 and Figure 2B). The significantly lower risk of clinical events in the intravascular imaging–guided group was associated with lower incidence of target vessel revascularization (3.0% vs 5.5%; HR, 0.55; 95% CI, 0.30-0.99; *P* = .046). Incidences of hard end points including cardiac death or target vessel–related MI were not significantly different between the 2 groups (eFigure 4 in Supplement 1). Multivariable analyses showed consistent results (Table 3). *P* for interaction between the presence of CKD and the use of intravascular imaging for the primary end point was .46, implying no significant interaction.

**Table 3.** Primary and Secondary End Points in Patients Without Chronic Kidney Disease

End point	Participants, No. (%)^a^			HR (95% CI)	
	Total (N = 1343)	Intravascular imaging–guided (n = 889)	Angiography-guided (n = 454)	Univariable analysis	Multivariable analysis^b^
Primary end point					
Target vessel failure	95 (7.6)	54 (6.4)	41 (9.9)	0.66 (0.44-0.99)	0.65 (0.43-0.99)
Secondary end point					
Target vessel failure without procedure-related MI	58 (4.9)	30 (3.7)	28 (7.0)	0.54 (0.32-0.90)	0.53 (0.31-0.89)
Cardiac death or target vessel–related MI	62 (4.8)	37 (4.4)	25 (5.6)	0.75 (0.45-1.25)	0.73 (0.44-1.22)
All-cause death	36 (3.4)	20 (2.8)	16 (4.4)	0.63 (0.33-1.21)	0.61 (0.31-1.19)
Cardiac death	15 (1.2)	7 (0.8)	8 (1.9)	0.44 (0.16-1.22)	0.40 (0.14-1.13)
MI					
Any	54 (4.2)	33 (3.9)	21 (4.9)	0.80 (0.46-1.38)	0.77 (0.44-1.34)
Target vessel–related MI	49 (3.8)	30 (3.6)	19 (4.2)	0.80 (0.45-1.43)	0.8 (0.44-1.40)
Spontaneous MI	10 (0.9)	5 (0.8)	5 (1.1)	0.50 (0.15-1.74)	0.52 (0.15-1.84)
Procedure-related MI	39 (2.9)	25 (2.8)	14 (3.1)	0.91 (0.47-1.75)	0.87 (0.45-1.70)
Nontarget vessel–related MI	6 (0.5)	3 (0.3)	3 (0.9)	0.51 (0.10-2.51)	0.40 (0.08-1.99)
Repeat revascularization	69 (6.2)	41 (5.5)	28 (7.5)	0.73 (0.45-1.19)	0.74 (0.45-1.20)
Target vessel revascularization	44 (3.8)	23 (3.0)	21 (5.5)	0.55 (0.30-0.99)	0.55 (0.30-1.00)
Target lesion revascularization	35 (3.1)	17 (2.2)	18 (4.8)	0.47 (0.24-0.91)	0.48 (0.25-0.94)
Nontarget lesion revascularization	35 (3.3)	21 (2.9)	14 (4.0)	0.75 (0.38-1.48)	0.77 (0.39-1.52)
Definite stent thrombosis	2 (0.1)	0	2 (0.4)	NA	NA
Contrast-induced nephropathy	24 (1.8)	17 (1.9)	7 (1.5)	1.36 (0.56-3.28)	1.40 (0.57-3.46)

Abbreviations: HR, hazard ratio; MI, myocardial infarction; NA, not applicable.

^a^ Percentages are 3-year Kaplan-Meier estimates.

^b^ Adjusted variables for multivariable analysis were age, sex, acute coronary syndrome, history of percutaneous coronary intervention, 3 or more complex coronary lesions, and use of adjunctive noncompliant balloon.

### Outcome Differences Between Intravascular Imaging- and Angiography-Guided PCI According to GFR

eFigure 5 in Supplement 1 presents the HR for the primary end point of intravascular imaging–guided PCI compared with angiography-guided PCI stratified by different classes of GFR. Although the advantages of intravascular imaging–guided PCI compared with angiography-guided PCI remained consistent across all stages of CKD, the greatest reduction of TVF in the intravascular imaging–guided PCI was found in patients with CKD stage 3 (GFR, ≤30 mL/min/1.73m^2^ to <60 mL/min/1.73m^2^) (8.8% vs 21.2%; HR, 0.28; 95% CI, 0.11-0.68; *P* = .02).

## Discussion

In this prespecified substudy of the RENOVATE-COMPLEX-PCI, we aimed to investigate whether the benefit of intravascular imaging–guided PCI differs according to the presence of CKD. A summary of the findings is as follows. First, patients with CKD had a 2-fold higher risk of TVF than those without CKD after PCI for complex coronary artery lesions. Second, the intravascular imaging guidance during complex PCI was associated with significantly reduced risk of TVF compared with angiography guidance, regardless of the CKD presence. The lower incidence of the primary end point was primarily due to a lower risk of cardiac death or target vessel–related MI in patients with CKD, but target vessel revascularization in those without CKD. Third, the greatest benefit of intravascular imaging guidance for TVF during complex PCI was shown in patients with stage 3 CKD (30≤GFR<60 mL/min/1.73m^2^).

CKD is a well-known risk factor for ischemic heart disease, not only for the high prevalence of concomitant cardiovascular risk factors, but also for the pathologic effect of uremia on the cardiovascular system.^1,18^ Coronary artery lesions in CKD are recognized for their heightened complexity, characterized by advanced atherosclerotic plaques with calcification. As a result, PCI for these types of lesions often requires more effort to optimize the procedural outcome.^3,4,19^ Furthermore, numerous studies have consistently shown that patients with concomitant coronary artery disease and CKD had poorer clinical outcomes following PCI than those without CKD.^20,21,22^ Similar to previous studies, in the current study, patients with CKD had more cardiovascular risk factors and presented with multivessel diseases more frequently. Procedural optimization by intravascular imaging was also more difficult to achieve in patients with CKD assigned to the intravascular imaging–guided PCI group. In addition, patients with CKD who underwent complex PCI were independently associated with a higher risk of adverse cardiovascular events than those without CKD. In agreement with a previous study assessing the association between GFR and risk of cardiovascular events in a community-based population,^23^ the current study showed a linear inverse association between baseline GFR and risk of TVF following complex PCI. These results all suggest that CKD is independently associated with prognosis after complex PCI. Thus, careful decision-making for the treatment of coronary artery lesions and additional efforts for reducing follow-up adverse events should be required in this population.

The use of intravascular imaging during PCI offers comprehensive anatomical insights into the coronary artery, facilitates optimal stent selection, and enhances the optimization of stent implantation. In addition, IVUS (not OCT) can suppress kidney function deterioration by minimizing contrast volume during PCI in patients with CKD, as has been suggested by several small studies.^24,25^ Theoretically, it is possible that the benefits of intravascular imaging guidance during complex PCI are more pronounced in patients with CKD, reducing the risk of CIN and adverse cardiovascular events. In the current study, patients with CKD who underwent intravascular imaging–guided PCI had a significantly lower risk of TVF than those who underwent angiography-guided PCI. Although the benefits of intravascular imaging guidance for patients with CKD were already presented in the Intravascular Ultrasound Guided Drug Eluting Stents Implantation in “All-Comers” Coronary Lesions (ULTIMATE) trial substudy,^10^ there were several new findings in the current study. First, unlike the ULTIMATE trial, which enrolled an all-comer population, the RENOVATE-COMPLEX-PCI, which enrolled complex coronary lesions only, could show greater benefits from the use of intravascular imaging. In this regard, intravascular imaging–guided PCI was associated with significantly lower risk of TVF even in the population without CKD. Second, the lower rate of TVF in the intravascular imaging guidance was mainly associated with a lower rate of hard end points in patients with CKD. This observation is somewhat different from the ULTIMATE trial substudy. Third, although there was no statistical difference due to the small sample size, intravascular imaging in CKD numerically reduced the risk of CIN without increase of used contrast amount even though OCT was used for some patients. These results emphasize the importance of using an imaging device in PCI for the CKD population, as the consequences of not using intravascular imaging for PCI could be more serious in those with CKD than those without CKD. However, because the small sample size of patients with CKD and the lack of control for various factors that may affect contrast volume, such as differences in the proportion of patients on dialysis and the proportion using OCT, it should be interpreted with caution. A larger, well-designed randomized trial is needed to evaluate the effects of intravascular imaging on the risk of CIN for patients with CKD.

Interestingly, we found that the clinical benefits of intravascular imaging guidance during complex PCI were greatest in patients with stage 3 CKD (GFR, >30 mL/min/1.73 m^2^ to <60 mL/min/1.73m^2^). This subgroup represents the population for which the most cautious control for the use of contrast should be applied due to well-remaining kidney function and the potential for deterioration. Accordingly, the current finding is meaningful in that careful procedures under intravascular imaging guidance for minimizing contrast volume might improve the clinical outcomes in patients with marginal kidney function. The nonsignificant difference in clinical outcomes between the 2 groups in those with GFR under 30mL/min/1.73m^2^ or receiving dialysis could have resulted from the underlying high risk of major cardiovascular events during follow-up, high enough to nullify the benefits of intravascular imaging. Considering these populations were either included at a small number or excluded by design in the previous and current studies on intravascular imaging,^6,10,26,27^ a larger study will be needed to confirm this finding.

### Limitations

This study has several limitations. First, an uneven proportion of choice for intravascular imaging devices resulted in only a small fraction of OCT, and its benefit and harm could not be accurately compared with IVUS or angiography alone. A relatively small CKD population also limits interpretation from the comparison of outcomes among the 3 groups. Therefore, this was a hypothesis-generating study. Second, as with the main study, masking of the operator was impossible due to the different study procedures for stent implantations. However, precisely defined criteria of angiography and imaging-based optimization, quantitative coronary angiography analysis at the core laboratories, and blinded clinical event adjudication was conducted to minimize the risk of potential bias. Third, as intravascular imaging-based measurement is impossible in the angiography-guided PCI group, the only available data to explain the differences in outcomes were data from quantitative coronary angiography. Fourth, as this was a substudy that was not dedicated to patients with CKD, mid- to long-term kidney-related outcomes were missing. In addition, there was not a specific protocol for reducing contrast volume using intravascular imaging in patients with CKD. Fifth, although bleeding is an important factor in patients with CKD that could have affected the adverse outcomes, these data were unavailable in the current study.

## Conclusions

In patients with complex coronary artery lesions, intravascular imaging–guided PCI was superior to angiography-guided PCI in reducing the risk of a composite of cardiac death, target vessel–related MI, or target vessel revascularization in both the CKD and the non-CKD population. The benefit was more apparent in the CKD population, particularly in those with stage 3 CKD (GFR, >30 mL/min/1.73 m^2^ to <60 mL/min/1.73m^2^).

## Notes

Supplement 1.
eMethods
**eFigure 1.** Study Flow **eFigure 2.** Proportion of IVUS and OCT Use According to the Presence of Chronic Kidney Disease **eFigure 3.** Histogram of Glomerular Filtration Rate **eTable 1.** Comparison of Baseline Characteristics According to the Presence of Chronic Kidney Disease **eTable 2.** Baseline Angiographic and Procedural Characteristics According to the Presence of Chronic Kidney Disease **eTable 3.** Lesion-Level Analysis of Quantitative Coronary Angiography According to CKD and Allocation Group **eTable 4.** Lesion-Level Analysis of Intravascular Imaging According to CKD **eFigure 4.** Comparison of 3-Year Cardiac Death or Target Vessel-Related Myocardial Infarction Between the Imaging-Guided and Angiography-Guided PCI, Stratified by the Presence of Chronic Kidney Disease **eFigure 5.** Risk of Target Vessel Failure in 2 Allocation Groups According to the Degree of Kidney Function
eReferences

Supplement 2.
Nonauthor Collaborators

Supplement 3.
Data Sharing Statement
