# Supplementary material for: Intravascular Imaging in Patients With Complex Coronary Lesions and Chronic Kidney Disease
Source: JAMA Netw Open. 2023 Nov 29;6(11):e2345554. doi: 10.1001/jamanetworkopen.2023.45554 (PMC10687657; doi:10.1001/jamanetworkopen.2023.45554)
Supplement: Supplement 1. — eMethods eFigure 1. Study Flow eFigure 2. Proportion of IVUS and OCT Use According to the Presence of Chronic Kidney Disease eFigure 3. Histogram of Glomerular Filtration Rate eTable 1. Comparison of Baseline Characteristics According to the Presence of Chronic Kidney Disease eTable 2. Baseline Angiographic and Procedural Characteristics According to the Presence of Chronic Kidney Disease eTable 3. Lesion-Level Analysis of Quantitative Coronary Angiography According to CKD and Allocation Group eTable 4. Lesion-Level Analysis of Intravascular Imaging According to CKD eFigure 4. Comparison of 3-Year Cardiac Death or Target Vessel-Related Myocardial Infarction Between the Imaging-Guided and Angiography-Guided PCI, Stratified by the Presence of Chronic Kidney Disease eFigure 5. Risk of Target Vessel Failure in 2 Allocation Groups According to the Degree of Kidney Function eReferences [file jamanetwopen-e2345554-s001.pdf]

Supplemental Online Content

Kwon W, Choi KH, Song YB et al. Intravascular imaging in patients with complex coronary lesions and chronic kidney disease. *JAMA Netw Open*. 2023;6(11):e2345554.
doi:10.1001/jamanetworkopen.2023.45554

eMethods

eFigure 1. Study Flow

eFigure 2. Proportion of IVUS and OCT Use According to the Presence of Chronic Kidney Disease

eFigure 3. Histogram of Glomerular Filtration Rate

eTable 1. Comparison of Baseline Characteristics According to the Presence of Chronic Kidney Disease

eTable 2. Baseline Angiographic and Procedural Characteristics According to the Presence of Chronic Kidney Disease

eTable 3. Lesion-Level Analysis of Quantitative Coronary Angiography According to CKD and Allocation Group

eTable 4. Lesion-Level Analysis of Intravascular Imaging According to CKD

eFigure 4. Comparison of 3-Year Cardiac Death or Target Vessel–Related Myocardial Infarction Between the Imaging-Guided and Angiography-Guided PCI, Stratified by the Presence of Chronic Kidney Disease

eFigure 5. Risk of Target Vessel Failure in 2 Allocation Groups According to the Degree of Kidney Function

eReferences

This supplemental material has been provided by the authors to give readers additional information about their work.

eMethods

Inclusion and Exclusion Criteria

Inclusion Criteria

① Subject must be at least 19 years of age
② Coronary artery disease requiring PCI
③ Patients with a complex lesion defined as:
   1) True bifurcation lesion (Medina 1,1,1/1,0,1/0,1,1) with side branch ≥2.5mm size
   2) Chronic total occlusion (≥3 months) as target lesion
   3) Unprotected LM disease PCI (LM ostium, body, distal LM bifurcation, including non-true bifurcation)
   4) Long coronary lesions (implanted stent ≥38 mm in length)
   5) Multi-vessel PCI (≥2 vessels treated at one PCI session)
   6) Multiple stents needed (≥3 more stent per patient)
   7) In-stent restenosis lesion as target lesion
   8) Severely calcified lesion (encircling calcium in angiography)
   9) Ostial coronary lesion (LAD, LCX, RCA)
④ Subject is able to verbally confirm understandings of risks, benefits and treatment alternatives of receiving invasive evaluation and PCI and he/she or his/her legally authorized representative provides written informed consent prior to any study related procedure.

Exclusion criteria

① Target lesions not amenable to PCI based on operators' review
② Cardiogenic shock (Killip class IV) at presentation
③ Intolerance to aspirin, clopidogrel, prasugrel, ticagrelor, heparin, or everolimus
④ Known true anaphylaxis to contrast medium (not allergic reaction but anaphylactic shock)
⑤ Pregnancy or breast feeding
⑥ Non-cardiac co-morbid conditions are present with life expectancy <1 year or that may result in protocol non-compliance (per site investigator's medical judgment)
⑦ Unwillingness or inability to comply with the procedures described in this protocol.

PCI denotes percutaneous coronary intervention, LM left main coronary artery, LAD left anterior descending artery LCX left circumflex artery, RCA right coronary artery.

© 2023 Kwon W et al. *JAMA Network Open.*

Primary and Secondary Endpoints

**Primary Endpoint**	
Target vessel failure	A composite of cardiac death, target vessel MI, and clinically-driven target vessel revascularization.
**Secondary Endpoints**	
Target vessel failure without procedure-related MI	
Cardiac death or target-vessel MI	
All-cause death	
Cardiac death	
Target vessel MI with procedure-related MI	
Target vessel MI without procedure-related MI	
Any MI with procedure-related MI	
Any MI without procedure-related MI	
Non-target vessel related MI	
Target lesion revascularization	
Target vessel revascularization	
Any revascularization (clinically-driven)	
Stent thrombosis	
Incidence of contrast-induced nephropathy	
Total amount of contrast use	
Total procedural time	
Total medical cost – not reported in this publication	

MI denotes myocardial infarction

© 2023 Kwon W et al. *JAMA Network Open.*

Supplementary Statistical Analysis

Hypothesis: An intravascular imaging-guided PCI strategy for patients with complex coronary artery lesions would reduce target vessel failure (a composite of cardiac death, target vessel myocardial infarction, and target vessel revascularization), compared with an angiography-guided PCI strategy.

Null hypothesis: An intravascular imaging-guided PCI strategy for patients with complex coronary artery lesions would not reduce target vessel failure (a composite of cardiac death, target vessel myocardial infarction, and target vessel revascularization), compared with an angiography-guided PCI strategy.

Reported Event Rates in Previous Studies of Complex PCI

Study	Sample Size	Timepoint	MACE		
			Intravascular Imaging-guided PCI	Angiography-guided PCI	Relative Risk Reduction, %
**ADAPT-DES[1]**	8665	1 Year	3.1%	4.7%	34.0%
**AVIO trial[2]**	284	2 Year	16.9%	23.2%	27.2%
**HOME DES IVUS[3]**	210	1.6 Years	11.0%	12.0%	8.3%
**RESET[4]**	543	1 Year	4.5%	7.3%	38.4%
**CTO-IVUS[5]**	402	1 Year	2.6%	7.1%	63.4%
**IVUS-XPL[6]**	1400	1 Year	2.9%	5.8%	50.0%

The current trial was designed as a superiority trial to follow enrolled patients until a prespecified follow-up duration of the last patient enrolled. Since the follow-up duration of the previous studies varied, we assumed that the annual incidence of target vessel failure in the angiography-guided PCI group would be 6.0%, based on the results of the CTO-IVUS, RESET, and IVUS-XPL studies. These 3 studies were selected because they were randomized trials conducted in South Korea and the follow-up duration was 1 year. As presented in the above table, the relative risk reduction of target vessel failure of the 3 studies ranged from 38.4% to 63.4%. To be conservative, we assumed that the relative risk reduction at 1 year would be 40% and, in turn, the annual incidence of target vessel failure in the intravascular imaging-guided PCI group would be 3.6%.

Sample Size Calculation

- Primary endpoint: Time to occurrence of target vessel failure (a composite of cardiac death, target vessel myocardial infarction, and target vessel revascularization)
- Assumed annual event rate of target vessel failure:
  - Intravascular imaging-guided PCI group (3.6%) vs. Angiography-guided PCI group (6%)
- Alpha = 0.05 (2-sided), β = 10%, Power (1- β) = 90%
- Accrual time: 3 years
- Total follow-up time: one year after last patient enrollment (median 2.5 years)

© 2023 Kwon W et al. *JAMA Network Open.*

- 2:1 Randomization
- Primary statistical method: Kaplan-Meier survival analysis with log-rank test
- Assumed dropout: total 5.0%

Based on the above assumptions, a total of 1620 patients (1080 and 540 patients for the intravascular imaging-guided group and the angiography-guided group, respectively) would be needed to evaluate the primary hypothesis with consideration of dropouts.

Consideration of 2:1 Randomization

Although previous randomized controlled trials were potentially limited by enrolling a small number of patients, limited follow-up duration, or enrolling patients with highly selected coronary artery lesion subsets, they consistently showed the potential benefit of intravascular imaging-guided PCI compared with angiography-guided PCI.[2,3,5,7,8] In this regard, the executive committee members tried to maximize the potential benefit of intravascular imaging-guided PCI in the treatment of complex coronary artery lesions. While we did not collect the exact proportion of PCI cases done with intravascular imaging guidance from all the participating centers, the adoption rate of intravascular imaging-guided PCI in Korea is about 27.5% to 28.6% according to the Korean Percutaneous Coronary Intervention (K-PCI) Registry that includes 92 participating centers.[9] Considering the adoption rate of intravascular imaging-guided PCI in Korea, a 2:1 randomization ratio should not introduce bias when interpreting the trial results.

Statistical Analysis

Continuous variables were presented as means ± standard deviations or median and interquartile range and were compared using Welch's t-test. Categorical variables are presented as number and relative frequency (%) and are compared using the Chi-square test. The assumption of proportional hazards model was evaluated using Schoenfeld residuals.

© 2023 Kwon W et al. *JAMA Network Open.*

Definition of Clinical Events

Death

Death as defined by the Academic Research Consortium is as follows:[10]

All death was considered to be cardiac death unless an unequivocal noncardiac cause can be established. Specifically, any unexpected death, even in patients with coexisting potentially fatal noncardiac disease (eg, cancer, infection), should be classified as cardiac. The cause of death (cardiac vs. non-cardiac) was adjudicated by an independent clinical events adjudication committee.

Cardiac death: Any death due to a proximate cardiac cause (eg, myocardial infarction, low-output failure, fatal arrhythmia), unwitnessed death and death of unknown cause, and all procedure-related deaths, including those related to concomitant treatment, was classified as cardiac death.

Non-cardiac death: Any death not covered by the above definitions, such as death caused by infection, malignancy, sepsis, pulmonary causes, accident, suicide, or trauma.

Myocardial Infarction

The definition of myocardial infarction used in this trial was based on the Third Universal Definition of Myocardial Infarction for spontaneous myocardial infarction,[11] and the Society for Cardiovascular Angiography and Interventions definition for procedure-related myocardial infarction.[12]

Spontaneous Myocardial Infarction

Myocardial infarction was considered to be present when there was evidence of myocardial necrosis in a clinical setting consistent with acute myocardial ischemia.[11] Under these conditions any one of the following criteria meets the diagnosis for myocardial infarction:

1) Detection of a rise and/or fall of cardiac troponin with at least one value above the 99th percentile upper reference limit and with at least one of the following:

- Symptoms of ischemia.
- New or presumed new significant ST-segment–T wave changes or new left bundle branch block (LBBB).
- Development of pathological Q waves in the ECG.
- Imaging evidence of new loss of viable myocardium or new regional wall motion abnormality.
- Identification of an intracoronary thrombus by angiography or autopsy.

2) Cardiac death with symptoms suggestive of myocardial ischemia and presumed new ischemic ECG changes or new LBBB, but death occurred before cardiac biomarkers were obtained, or before cardiac biomarker values would be increased.

© 2023 Kwon W et al. *JAMA Network Open.*

3) Stent thrombosis associated with myocardial infarction when detected by coronary angiography or autopsy in the setting of myocardial ischemia and with a rise and/or fall of cardiac biomarker values with at least one value above the 99th percentile upper reference limit.

Procedure-Related Myocardial Infarction

Procedure-related myocardial infarction is defined as follows:[12]

1) In patients with normal baseline CK-MB, myocardial infarction was considered to have occurred when the peak CK-MB measured within 48 hours of the procedure rises to at least 10 times the local laboratory upper reference limit; or to at least 5 times the upper reference limit with new pathologic Q-waves in at least 2 contiguous leads or new persistent LBBB; or in the absence of CK-MB measurements and a normal baseline cardiac troponin (cTn), a cTn (I or T) level measured within 48 hours of the PCI rises to at least 70 times the local laboratory upper reference limit; or at least 35 times upper reference limit with new pathologic Q-waves in at least 2 contiguous leads, or new persistent LBBB.

2) In patients with an elevated baseline CK-MB (or cTn) in whom the biomarkers are stable or falling, the definition was based on when CK-MB (or cTn) rises by an absolute increment equal to those levels recommended above from the most recent pre-procedure level.

3) In patients with an elevated baseline CK-MB (or cTn) in whom the biomarker levels have not been shown to be stable or falling, the definition is based on when CK-MB (or cTn) rises by an absolute increment equal to those levels recommended above plus new ST-segment elevation or depression plus signs consistent with a clinically relevant MI, such as new onset or worsening heart failure or sustained hypotension.

Revascularization

A coronary revascularization procedure may be either a PCI or a coronary artery bypass grafting (CABG) surgery. Revascularization is defined by the Academic Research Consortium[10] as follows:

The coronary segments that were revascularized were sub-classified as:

Target Lesion: A target lesion was defined as a lesion revascularized in the index procedure (or during a planned or provisional staged procedure). The left main target lesion extends from the left main stem ostium to the end of the 5 mm proximal segments of the left anterior descending and left circumflex arteries as well as the ramus intermedius if the latter vessel has a vessel diameter of at least 2 mm.

Target Vessel: The target vessel was defined as the entire major coronary vessel proximal and distal to the target lesion including upstream and downstream branches and the target lesion itself. The left main coronary artery and any vessel originating from the left main coronary artery, or its major branches is, by definition, considered a target vessel for the purposes of this trial.

© 2023 Kwon W et al. *JAMA Network Open.*

Target Vessel Non-Target Lesion: The target vessel non-target lesion was a lesion in the epicardial vessel or branch or graft that contains the target lesion; however, this lesion is outside of the target lesion by at least 5 mm distal or proximal to the target lesion determined by quantitative coronary angiography.

Non-Target Vessel: The non-target vessel was any vessel that was not attempted to be revascularized at the index procedure but was subsequently revascularized.

Target Lesion Revascularization: Target lesion revascularization was defined as any repeat PCI of the target lesion or bypass surgery of the target vessel performed for restenosis or other complication of the target lesion. All target lesion revascularizations were classified prospectively as clinically indicated or not clinically indicated by the investigator prior to repeat angiography. An independent angiographic core laboratory verified that the severity of the percent diameter stenosis met the requirements for clinical indication and overruled cases where investigator reports were not in agreement. The target lesion was defined as the treated segment from 5 mm proximal to the stent and to 5 mm distal to the stent.

Target Vessel Revascularization: Target vessel revascularization was defined as any repeat percutaneous intervention or surgical bypass of any segment of the target vessel. The target vessel was defined as the entire major coronary vessel proximal and distal to the target lesion, which included upstream and downstream branches and the target lesion itself.

Non-Target Lesion Revascularization: Any revascularization in a lesion other than the target lesion was considered a non-target lesion revascularization.

Non-Target Vessel Revascularization: Any revascularization in a vessel other than the target vessel was considered a non-target vessel revascularization.

All revascularization events were adjudicated as either clinically driven or non-clinically driven. Revascularization was considered clinically driven if the diameter stenosis of the revascularized coronary segment is at least 50% by quantitative coronary angiography and any of the following criteria for ischemia were met:

- A positive functional study corresponding to the area served by the target lesion; or
- Ischemic ECG changes at rest in a distribution consistent with the target vessel; or
- Typical ischemic symptoms referable to the target lesion; or
- Positive invasive physiologic test (fractional flow reserve ≤0.80 or instantaneous wave-free ratio ≤0.89); or
- Presence of stenosis with at least 70% diameter stenosis, even in the absence of other criteria

© 2023 Kwon W et al. *JAMA Network Open.*

Stent Thrombosis

Stent thrombosis was defined by the Academic Research Consortium[10] as follows:

1) Timing: a) Acute b) Subacute c) Late, and d) Very late

Acute stent thrombosis*	0-24 hours after stent implantation
Subacute stent thrombosis*:	More than 24 hours to 30 days after stent implantation
Late stent thrombosis†:	More than 30 days to 1-year after stent implantation
Very late stent thrombosis†:	More than 1-year after stent implantation

* Acute/subacute can also be replaced by early stent thrombosis. Early stent thrombosis (0 to 30 days) is currently used to define stent thrombosis occurring from day 0 to day 30 by the international interventional cardiology community.

†This definition includes "primary" as well as "secondary" late stent thrombosis; "secondary" late stent thrombosis was defined as stent thrombosis that occured after a target segment revascularization.

2) Stent Thrombosis Categories: a) Definite b) Probable, and c) Possible

Definite stent thrombosis: Definite stent thrombosis was considered to have occurred by either angiographic or pathologic confirmation.

Angiographic confirmation of stent thrombosis: The presence of an intracoronary thrombus that originates in the stent or in the segment 5 mm proximal or distal to the stent and presence of at least 1 of the following criteria within a 48-hour time window:

- Acute onset of ischemic symptoms at rest; or
- New ischemic ECG changes that suggest acute ischemia; or
- Typical rise and fall in cardiac biomarkers (refer to the definition of spontaneous myocardial infarction); or
- Nonocclusive thrombus: Intracoronary thrombus was defined as a (spheric, ovoid, or irregular) noncalcified filling defect or lucency surrounded by contrast material (on 3 sides or within a coronary stenosis) seen in multiple projections at the time of coronary angiography, or persistence of contrast material within the lumen, or a visible embolization of intraluminal material downstream.
- Occlusive thrombus: Was defined as Thrombolysis in Myocardial Infarction (TIMI) Grade 0 flow (no flow of contrast after the thrombotic stenosis) or TIMI Grade 1 flow (flow past the thrombotic stenosis that doesn't fill the vessel entirely) within the stent or proximal to a stent up to the most adjacent proximal side branch or main branch (if the stent originates from the side branch).

Pathological confirmation of stent thrombosis: Evidence of recent thrombus within the stent

© 2023 Kwon W et al. *JAMA Network Open.*

determined at autopsy or via examination of tissue retrieved following thrombectomy.

[2] Probable stent thrombosis: The clinical definition of probable stent thrombosis was considered to have occurred after intracoronary stenting in the following cases:

- Any unexplained death within the first 30 days; or
- Irrespective of the time after the index procedure, any myocardial infarction that was related to documented acute ischemia in the territory of the implanted stent without angiographic confirmation of stent thrombosis and in the absence of any other obvious cause.

[3] Possible stent thrombosis: The clinical definition of possible stent thrombosis was considered to have occurred with any unexplained death from 30 days after intracoronary stenting until the end of trial follow-up.

Contrast-Induced Nephropathy

Contrast-induced nephropathy was defined as an increase in serum creatinine of at least 0.5mg/dL or at least 25% from baseline within 48-72 hours after exposure to a contrast agent.[10]

© 2023 Kwon W et al. *JAMA Network Open.*

Protocol for Intravascular Imaging Device Use and Angiography-guided PCI

PCI was performed using standard techniques. The drug-eluting stents used were either biodegradable polymer-coated everolimus eluting stents (Synergy, Boston Scientific Corporation, San Jose, CA, USA) or biocompatible polymer-coated everolimus-eluting stents (Xience, Abbott Vascular, St. Paul, MN, USA). The trial limited stent choice to these drug-eluting stents due to the well-validated efficacy and safety profile of biodegradable polymer-coated everolimus eluting stents and biocompatible polymer-coated everolimus-eluting stents,[13] the fact that these two stents have the highest market share in Korea, and the availability of these drug-eluting stents in all participating centers.

For patients assigned to intravascular imaging-guided PCI, the choice of intravascular imaging device (IVUS or OCT) was at the operators' discretion. While use of intravascular imaging was allowed at any step of the PCI procedure (prior to intervention, during PCI, and after stent implantation or angioplasty when performed as a standalone procedure), intravascular imaging evaluation after PCI was mandated for optimization of the stented segment.

Standard protocols for image acquisition were used with the IVUS (Opticross™, Boston Scientific Corporation, San Jose, CA, USA) or OCT (Dragonfly™, Abbott Vascular, St. Paul, MN, USA) devices. Before advancing the intravascular imaging catheter, intracoronary nitroglycerin (100 to 200 μg) was administered. For IVUS, the transducer was pulled back automatically at a speed of 0.5 mm per sec. For OCT, preheated contrast media at 37 °C was flushed through the guiding catheter at a rate of 2 to 4 ml per second for approximately 3 to 6 seconds by using an injector pump to obtain the OCT images. However, the final choice of pullback speed for the IVUS device and the injection rate and the amount of contrast media used during OCT imaging was also left to the operators' discretion. In case of a staged procedure during the same hospitalization, it was strongly recommended that the operator follow the initially allocated imaging or angiography guidance strategy.

Protocols for selecting the reference segments for the lesion, for selecting the appropriate size of the stent, and for stent optimization were prespecified based on previous reports in the literature.[14] In brief, proximal and distal reference sites were determined at cross-sections adjacent to the target lesion (at least 5 mm apart) that have the largest lumen and a plaque burden of less than 50% by IVUS. Using OCT, proximal and distal reference sites were determined at cross-sections adjacent to the target lesion (at least 5 mm apart) that have the most normal appearance and are free of lipid-containing plaque. The criteria used to determine optimal stent expansion were residual angiographic diameter stenosis (defined by percent diameter stenosis; ([mean reference vessel diameter – minimum lumen diameter]/mean reference vessel diameter) x 100) less than 10% and minimum stent area (defined by the lumen area measured by intravascular imaging devices at the site of the narrowest lumen inside of the stented segment) greater than 80% of the average reference lumen area or absolute minimum stent area greater than 5.5 mm^2 by IVUS or 4.5 mm^2 by OCT for a stenosis, except if the lesion was in the left main coronary artery. For a left main stenosis, an absolute minimal stent area greater than 7 mm^2 for distal left main coronary artery and greater than 8 mm^2 for the proximal left main coronary artery were used as optimization criteria, respectively.[14]

© 2023 Kwon W et al. *JAMA Network Open.*

An optimized procedural result in the intravascular imaging-guided PCI group was defined as sufficient stent expansion without major stent malapposition and edge dissection. Specific definitions are provided in the table below.

Major stent malapposition was defined as an acute malapposition with the distance between the vessel wall and the stent of at least 0.4 mm with longitudinal length of more than 1 mm. Major edge dissection was defined as a dissection occurring within 5 mm from the edge of the stent, extending to the medial layer with a dissection angle of at least 60° of the circumference of the vessel or at least 3 mm in length of dissection flap. If one of the above findings were identified by the intravascular imaging devices, additional procedures including adjunctive post-dilatation or additional stent implantation followed by further intravascular imaging were recommended to optimize the final results.

To avoid perforation, the non-compliant balloon diameter was recommended to be no larger than the nearest reference vessel diameter, or up to 0.5 mm larger than the mean reference lumen diameter after PCI, based on findings from the intravascular imaging. The maximal inflation pressure of the non-compliant balloon was left up to the operator; however, it was recommended that the non-compliant balloon was inflated to a pressure above the nominal rated pressure for the balloon. In case a major edge dissection was identified by intravascular imaging, additional stent implantation was recommended; the stent size selected was based on findings from the intravascular imaging study. After additional procedural optimization, the intravascular-imaging study was to be repeated until the acquisition of the optimized results, as described above. However, operators could decide to consider the procedure finished if it was believed that there was the potential risk for a complication associated with additional procedural optimization interventions.

	**IVUS**	**OCT**
**Reference Sites**	Largest size vessel lumen;<br>Plaque burden <50%;<br>At least 5 mm away from the target lesion	Most normal looking segment;<br>No lipid-containing plaque;<br>At least 5 mm away from the target lesion
**Stent Sizing**	Vessel diameter (external elastic membrane) is measured at the proximal and distal reference sites. The averaged value of the proximal and distal reference external elastic membrane diameter is used to determine the stent diameter.	Vessel diameter is measured at the distal reference sites (in cases where ≥180° of the external elastic membrane can be identified). Stent diameter is determined using the mean external elastic membrane diameter at the distal reference, rounded down to the nearest 0.25 mm. For example, if the mean external elastic membrane reference diameter is measured as 3.15 mm, then a 3.0 mm stent diameter will be

© 2023 Kwon W et al. *JAMA Network Open.*

	IVUS	OCT
		selected.<br>OR<br>The lumen diameter is measured at the distal reference sites (in cases where ≥180° of the external elastic membrane cannot be identified). Stent diameter is determined using the mean lumen diameter at the distal reference, rounded up to the nearest 0.25 mm. For example, if the mean distal reference lumen diameter is 2.55 mm, then a 2.75 mm stent diameter will be selected.
**Stent Length**	By measuring the distance from the distal to the proximal reference site.	
**Stent Optimization**		
● **Stent Expansion**	Visually assess that the residual angiographic diameter stenosis is <10% "AND"<br>● Non-left main coronary artery lesions: In-stent minimal stent lumen area > 80% of the average reference lumen area "OR" a minimal stent area of >5.5 mm^2 by IVUS and >4.5 mm^2 by OCT.<br>● Left main coronary artery lesions: Minimal stent luminal area of >7 mm^2 for a distal left main coronary artery stenosis and >8 mm^2 for a proximal left main coronary artery stenosis by IVUS.	
● **Stent Apposition**	No major malapposition (defined as an acute malapposition of ≥0.4 mm with longitudinal extension >1 mm) of the stent over its entire length against the vessel wall.	
● **Edge Dissection**	No major edge dissection in the proximal or distal reference segments, defined as a location that is 5 mm from the edge of the stent, extends to the medial layer with potential to provoke flow disturbances (defined as ≥60° of the circumference of the vessel at the site of a dissection or ≥3 mm in length of the dissection flap)	
**Stent optimization technique**	**If any of above findings are identified, additional procedural intervention, including additional post-dilatation of the stent or additional stent implantation is recommended.**<br><br>For additional post-dilatation of the stent, the diameter of the non-compliant balloon should not be larger than the IVUS or OCT	

© 2023 Kwon W et al. *JAMA Network Open.*

	IVUS	OCT
	determined mean reference external elastic membrane diameter assessed after stenting of one or both segments (proximal or distal), or if the external elastic membrane cannot be measured, no more than 0.5 mm larger than the mean reference segment lumen diameter of one or both segments (proximal or distal) nearest to the dilation site.	

Among patients assigned to the angiography-guided PCI group, stent optimization was assessed and performed based on angiographic findings. A stent was considered optimized if the angiographic residual diameter stenosis is less than 10% by visual estimation and there was no flow limiting dissection (type C through F dissection). When underexpansion of the stent was suspected based on angiography, adjunctive balloon dilatation using non-compliant balloons was recommended. To avoid perforation, the non-compliant balloon diameter was recommended to be no larger than the nearest reference vessel diameter, or up to 0.5 mm larger than the mean reference lumen diameter after PCI. The maximal inflation pressure of the non-compliant balloon was left to the operator's discretion; however, it was recommended that the non-compliant balloon inflation was pressure was at least above the nominal rated pressure of the balloon. Additional procedural optimization was recommended until the optimized results (as described above) were obtained. Operators could decide to consider the procedure finished if it was believed that there was the potential risk for a complication associated with additional procedural optimization interventions.

After the index PCI procedure, dual antiplatelet therapy was recommended for at least 3 to 6 months in patients with stable ischemic heart disease and 6 to 12 months in those with an acute coronary syndrome, regardless of allocated arms.[15,16] However, the loading, maintenance dose, and duration of dual antiplatelet therapy were left up to the physicians' discretion. Regardless of the patient assignment, guideline directed medical therapy was recommended according to the current American College of Cardiology/ American Heart Association/ Society of Coronary Angiographers and Interventionalists or the European Society of Cardiology/European Association for Cardiothoracic Surgery guidelines.[17,18] All coronary angiograms and intravascular imaging data were analyzed by the independent core laboratories.

© 2023 Kwon W et al. *JAMA Network Open.*

Supplementary Tables

eTable 1. Comparison of Baseline Characteristics According to the Presence of Chronic Kidney Disease

	CKD (N=296)	Non-CKD (N=1343)	P Value
Age, years	70.3 (9.4)	64.5 (10.1)	<.001
Sex			
Male, n (%)	219 (74.0%)	1081 (80.5%)	.02
Female, n (%)	77 (26.0%)	262 (19.5%)	
Initial presentation, no. (%)			.005
Stable ischemic heart disease	155 (52.4%)	652 (48.5%)	
Acute coronary syndrome	141 (47.6%)	691 (51.5%)	
Medical history, no. (%)			
Hypertension	232 (78.4%)	773 (57.6%)	<.001
Diabetes mellitus	145 (49.0%)	472 (35.1%)	<.001
Dyslipidemia	145 (49.0%)	695 (51.7%)	.43
Current smoking	46 (15.5%)	261 (19.4%)	.14
Previous percutaneous coronary intervention	93 (31.4%)	302 (22.5%)	.001
Previous myocardial infarction	33 (11.1%)	84 (6.3%)	.005
Previous stroke	29 (9.8%)	83 (6.2%)	.04
Peripheral vascular disease	17 (5.7%)	27 (2.0%)	.001
Left ventricular ejection fraction, %	52.9 (14.0)	60.0 (10.5)	<.001
Creatinine, mg/dL, mean (SD)	2.9 (5.3)	0.8 (0.2)	<.001
Creatinine, mg/dL, median (Q1-Q3)*	1.4 (1.2-2.0)	0.8 (0.7-1.0)	<.001
Estimated glomerular filtration rate, mL/min/1.73m^2	41.6 (19.4)	89.7 (20.7)	<.001
Dialysis	52 (17.6%)	0 (0.0%)	<.001

Data presented as mean (standard deviation), median (interquartile range), or as n (%).
*P value calculated by comparing the two creatinine values as the factors following non-normal distribution.
Abbreviations: ACE, Angiotensin-converting enzyme; ARB, Angiotensin receptor blocker; CKD, chronic kidney disease; GFR, glomerular filtration rate; LV, left ventricle; NA, not applicable; PCI, percutaneous coronary intervention; SD, standard deviation.

© 2023 Kwon W et al. *JAMA Network Open.*

eTable 2. Baseline Angiographic and Procedural Characteristics According to the Presence of Chronic Kidney Disease

Characteristics	CKD (N=296)	Non-CKD (N=1343)	P Value
**Target lesion characteristics**			
Complex coronary lesions			
True bifurcation (Medina 1,1,1 / 1,0,1 / 0,1,1)	55 (18.6%)	304 (22.6%)	.15
Chronic total occlusion (≥3 months of occlusion)	58 (19.6%)	261 (19.4%)	.99
Unprotected left main disease	38 (12.8%)	154 (11.5%)	.57
Long coronary lesion (implanted stent length≥38 mm)	172 (58.1%)	726 (54.1%)	.23
Multivessel PCI (≥2 major coronary arteries treated)	114 (38.5%)	508 (37.8%)	.88
Multiple stents implanted (≥3 more stent per patient)	60 (20.3%)	245 (18.2%)	.47
In-stent restenosis lesion	59 (19.9%)	177 (13.2%)	.004
Severely calcified lesion (encircling calcium in angiography)	48 (16.2%)	183 (13.6%)	.29
Ostial coronary lesion	45 (15.2%)	206 (15.3%)	.99
Number of complex coronary lesions ≥ 3, no. (%)	98 (33.1%)	407 (30.3%)	.38
Arteries with stenosis, no. (%)			.002
1 vessel disease	70 (23.6%)	456 (34.0%)	
2 vessel disease	121 (40.9%)	500 (37.2%)	
3 vessel disease	105 (35.5%)	387 (28.8%)	
**Procedural characteristics**			
Total no. of target lesions treated	1.5 (0.8)	1.5 (0.7)	.25
Radial access, no. (%)	174 (58.8%)	1079 (80.3%)	<.001
Intravascular imaging devices used, no. (%)			<.001
Intravascular ultrasound	170 (57.4%)	643 (47.9%)	
Optical coherence tomography	28 (9.5%)	250 (18.6%)	
Not done	98 (33.1%)	450 (33.5%)	
Adjunctive non-compliant balloon used, no. (%)	185 (62.5%)	949 (70.7%)	.007
Rotablator used, no. (%)	10 (3.4%)	43 (3.2%)	.99

© 2023 Kwon W et al. *JAMA Network Open.*

Treatment devices used, no. (%)			<.001
Drug-eluting stent	283 (95.6%)	1311 (97.6%)	
Drug-coated balloon angioplasty	13 (4.4%)	32 (2.4%)	
Total no. of devices used per patient	2.0 (1.1)	1.9 (1.0)	.14
Dimensions of devices, mm			
Mean diameter	3.1 (0.4)	3.1 (0.4)	.61
Total length	58.2 (35.5)	55.0 (31.9)	.15
Volume of contrast media used, ml	194.7 (118.7)	210.1 (115.9)	.04
Procedural time, min	68.0 (48.0-90.0)	63.0 (46.0-88.0)	.12
Procedural success, no. (%)	291 (98.3%)	1337 (99.6%)	.048
Successful imaging-guided optimization*	73 (36.0%)	423 (47.6%)	.003

Data presented as mean (standard deviation), median (interquartile range), or as n (%).

*This analysis was available in imaging-guided group only (203 patients and 887 patients of CKD and non-CKD population, respectively).

Abbreviations: CKD, chronic kidney disease; PCI, percutaneous coronary intervention

© 2023 Kwon W et al. *JAMA Network Open.*

eTable 3. Lesion-Level Analysis of Quantitative Coronary Angiography According to CKD and Allocation Group

Characteristics	CKD (N=454)			Non-CKD (N=1984)			P Value (CKD vs. Non-CKD)
	Imaging-guided (N=305)	Angiography-guided (N=149)	P Value	Imaging-guided (N=1318)	Angiography-guided (N=666)	P Value	
**Location of target vessel**			.32			.42	.45
Left main artery	29 (9.5%)	19 (12.8%)		135 (10.2%)	54 (8.1%)		
Left anterior descending artery	125 (41.0%)	68 (45.6%)		576 (43.7%)	308 (46.2%)		
Circumflex artery	59 (19.3%)	21 (14.1%)		254 (19.3%)	130 (19.5%)		
Right coronary artery	92 (30.2%)	41 (27.5%)		353 (26.8%)	174 (26.1)		
**Quantitative coronary angiography**							
Pre-PCI QCA							
Proximal RD, mm	3.2 (0.5)	3.1 (0.5)	.16	3.2 (0.5)	3.1 (0.5)	<.001	.97
Distal RD, mm	2.7 (0.5)	2.7 (0.5)	.68	2.7 (0.5)	2.7 (0.4)	.35	.73
Minimum lumen diameter, mm	0.4 (0.4)	0.5 (0.4)	.52	0.4 (0.4)	0.4 (0.4)	.51	.31
Diameter stenosis, %	85.3 (12.4)	83.9 (13.1)	.29	85.5 (11.3)	85.5 (11.3)	.95	.31
Lesion length, mm	29.4 (16.2)	26.8 (13.5)	.08	28.2 (15.8)	26.8 (15.1)	.08	.32
Post-PCI QCA							
Minimum lumen diameter, mm	2.8 (0.5)	2.7 (0.5)	.45	2.8 (0.5)	2.7 (0.5)	.005	.48
Diameter stenosis, %	10.2 (10.3)	10.2 (8.2)	.94	9.7 (8.6)	9.9 (8.7)	.54	.35

Data presented as mean (standard deviation) or as number and percentage.

Abbreviations: CKD, chronic kidney disease; PCI, percutaneous coronary intervention; QCA, quantitative coronary angiography; RD, reference diameter.

© 2023 Kwon W et al. *JAMA Network Open.*

eTable 4. Lesion-Level Analysis of Intravascular Imaging According to CKD

Characteristics	CKD			Non-CKD			Total P Value (CKD vs. Non-CKD)
	Total (N=286)	IVUS (N=251)	OCT (N=35)	Total (N=1263)	IVUS (N=937)	OCT (N=326)	
**Pre-PCI**							
Proximal lumen area of reference, mm^2	10.0 (3.8)	10.3 (3.9)	8.4 (2.8)	9.9 (3.7)	10.5 (3.8)	8.4 (3.0)	.74
Distal lumen area of reference, mm^2	6.6 (2.8)	6.6 (2.8)	6.3 (2.2)	6.6 (2.8)	6.9 (2.8)	5.7 (2.6)	.89
Minimal lumen area, mm^2	2.2 (0.8)	2.3 (0.8)	1.6 (0.7)	2.2 (1.0)	2.4 (0.9)	1.8 (1.1)	.77
Plaque burden of minimal lumen, %	81.3 (6.8)	81.3 (6.8)	NA	80.5 (7.3)	80.8 (7.1)	72.8 (10.2)	.19
**Post-PCI**							
Minimal stent area, mm^2	5.5 (1.9)	5.5 (1.9)	5.8 (1.9)	5.7 (2.1)	5.8 (2.1)	5.2 (1.9)	.23
Expansion index, %	69.9 (16.5)	69.1 (16.1)	76.6 (19.0)	73.2 (17.7)	72.3 (17.3)	75.7 (18.7)	.007
Optimal stent expansion*	164 (57.3%)	135 (53.8%)	29 (82.9%)	829 (65.6%)	587 (62.6%)	242 (74.2%)	.01
Malapposition	19 (6.6%)	10 (4.0%)	9 (25.7%)	105 (8.3%)	41 (4.4%)	64 (19.6%)	.41
Major malapposition†	8 (2.8%)	4 (1.6%)	4 (11.4%)	33 (2.6%)	6 (0.6%)	27 (8.3%)	.99
Edge dissection	16 (5.6%)	7 (2.8%)	9 (25.7%)	67 (5.3%)	29 (3.1%)	38 (11.7%)	.96
Major edge dissection‡	3 (1.0%)	0 (0%)	3 (8.6%)	16 (1.3%)	7 (0.7%)	9 (2.8%)	.99

Data presented as mean (standard deviation) or as number and percentage.

*Optimal stent expansion was defined as a residual angiographic diameter stenosis of <10% and an in-stent minimum stent area of >80% of the average reference lumen area or an absolute in-stent minimum stent area of >5.5 mm^2 by IVUS imaging and >4.5 mm^2 by OCT imaging. For a left main coronary artery stenosis, optimal stent expansion was defined as an in-stent minimum stent area of >7 mm^2 for the distal left main coronary artery and >8 mm^2 for the proximal left main coronary artery was used as optimization criteria.

†Major malapposition was defined as an acute stent malapposition of ≥0.4 mm with longitudinal extension >1 mm of the stent over its entire length against the vessel wall.

‡Major edge dissection was defined as a dissection that occurred 5mm from the edge of the stent, extended to medial layer with potential to create a flow disturbance (defined as ≥60° of the circumference of the vessel at the site of dissection and/or ≥3 mm in length of a dissection flap).

Abbreviations: CKD, chronic kidney disease; IVUS, intravascular ultrasound; NA, not applicable; OCT, optical coherence tomography; PCI, percutaneous coronary intervention.

© 2023 Kwon W et al. *JAMA Network Open.*

Supplementary Figures

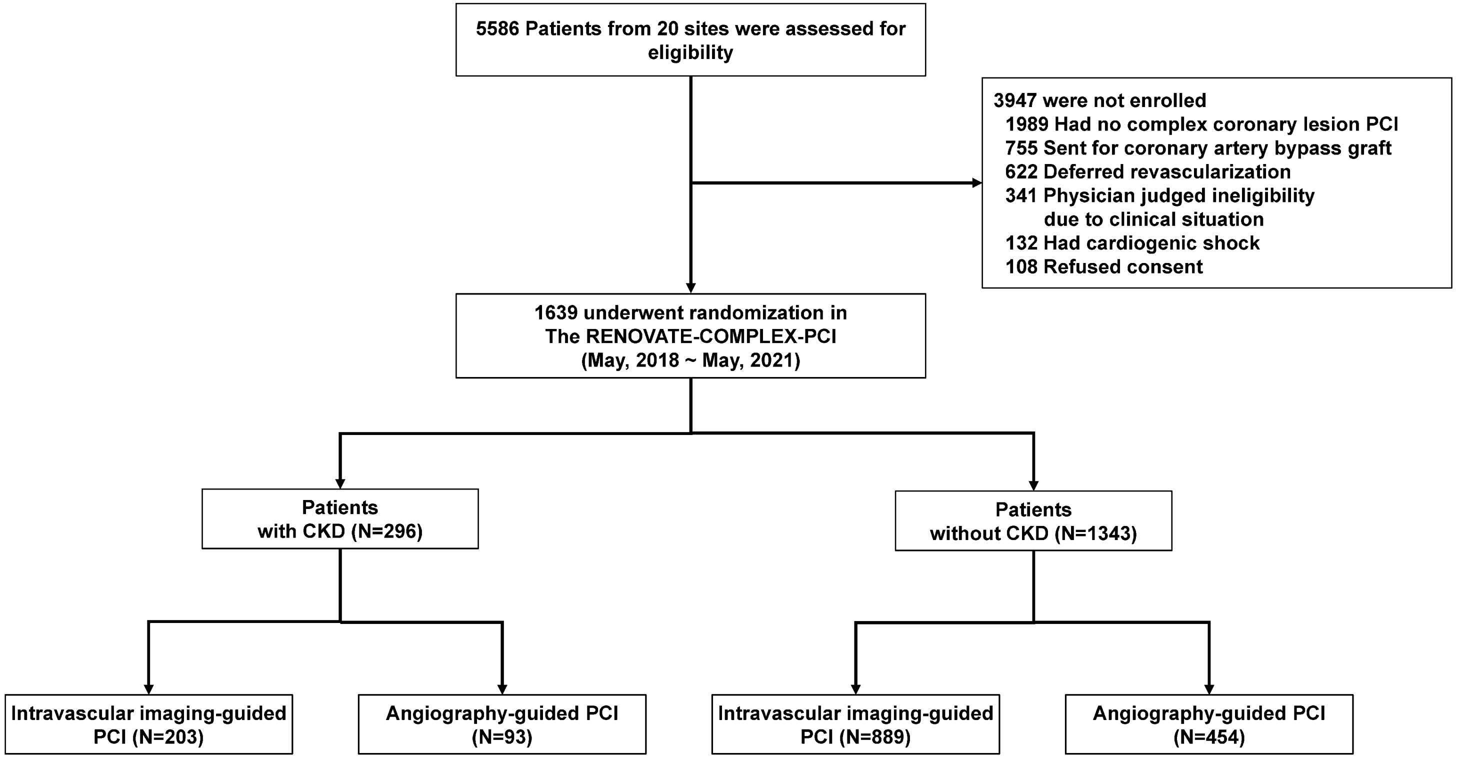

eFigure 1. Study Flow

Abbreviations: CKD, chronic kidney disease; PCI, percutaneous coronary intervention.

© 2023 Kwon W et al. *JAMA Network Open.*

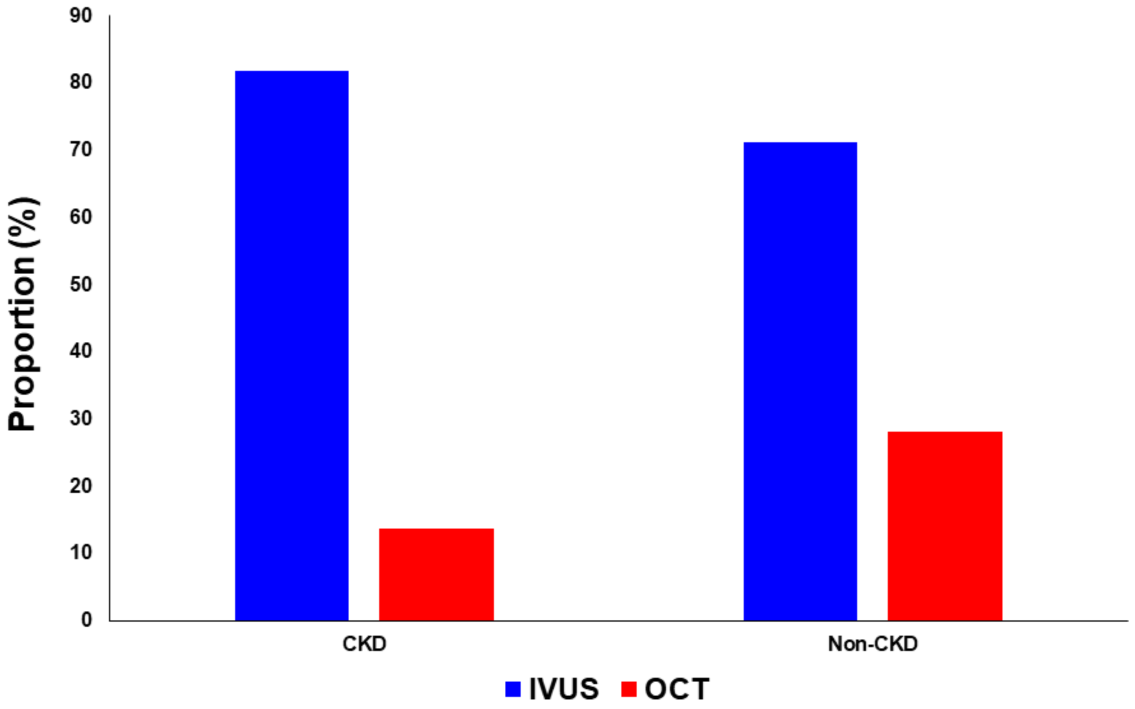

eFigure 2. Proportion of IVUS and OCT Use According to the Presence of Chronic Kidney Disease

There was significant difference of proportion of IVUS or OCT according to the presence of CKD (P<.001)

Abbreviations: IVUS, intravascular ultrasound; OCT, optical coherence tomography.

© 2023 Kwon W et al. *JAMA Network Open.*

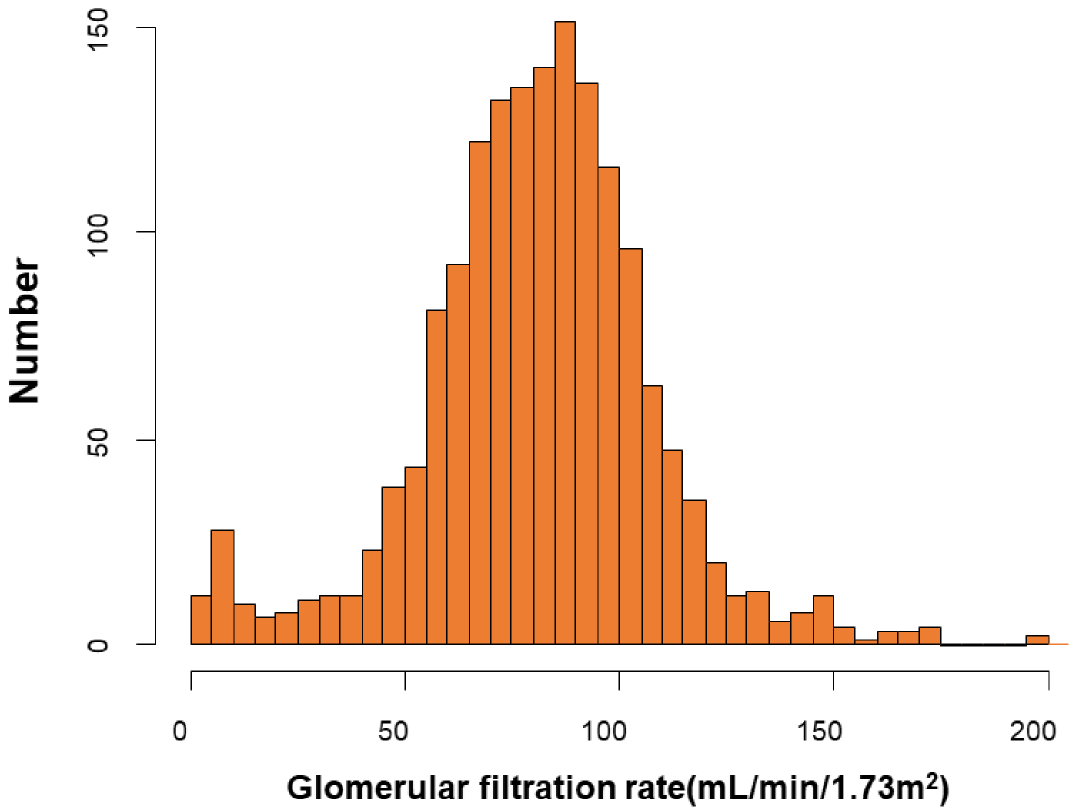

eFigure 3. Histogram of Glomerular Filtration Rate

Distribution of glomerular filtration rate is presented.

© 2023 Kwon W et al. *JAMA Network Open.*

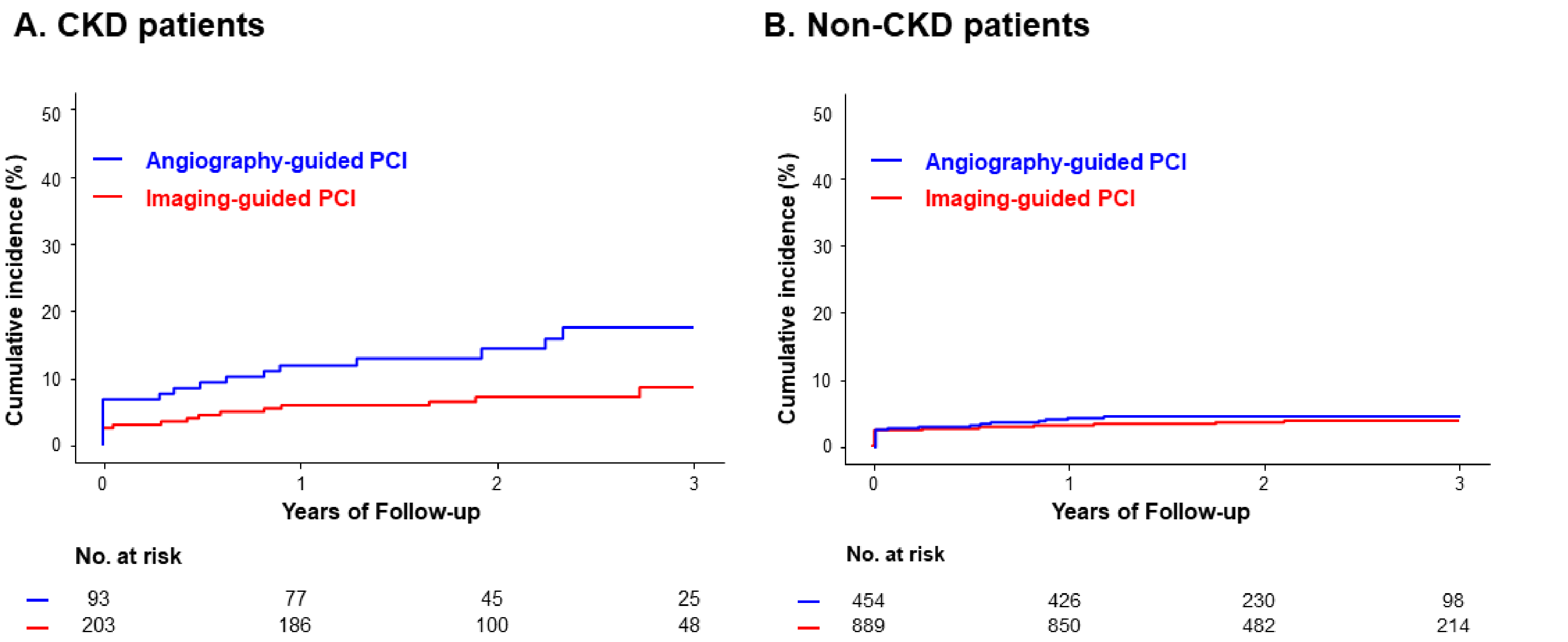

eFigure 4. Comparison of 3-Year Cardiac Death or Target Vessel-Related Myocardial Infarction Between the Imaging-Guided and Angiography-Guided PCI, Stratified by the Presence of Chronic Kidney Disease

Incidence of cardiac death or target vessel-related myocardial infarction was significantly higher in angiography-guided PCI group in CKD population (HR 0.39, 95% CI 0.20-0.76, P=.006), but not in non-CKD population (HR 0.75, 95% CI 0.45-0.125, P=.27)

Abbreviation. CI, confidence interval; CKD, chronic kidney disease; HR, hazard ratio; PCI, percutaneous coronary intervention

© 2023 Kwon W et al. *JAMA Network Open.*

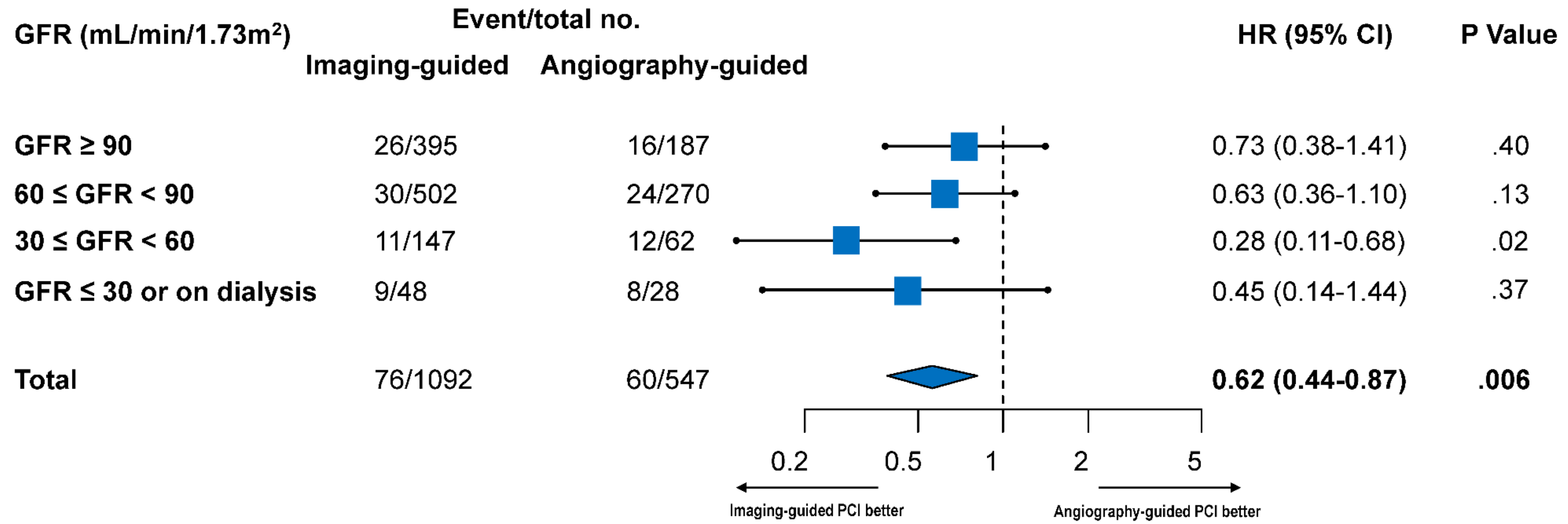

eFigure 5. Risk of Target Vessel Failure in 2 Allocation Groups According to the Degree of Renal Function

The cumulative incidence and HR with 95% CI of the primary endpoint are presented between intravascular imaging-guided and angiography-guided PCI according to the degree of renal function. Note that as chronic kidney disease population was defined as having GFR≤60 mL/min/1.73m^2 or having diagnosed chronic kidney disease in the past, some of the study population are actually included in GFR≥60 mL/min/1.73m^2 subgroup.

Abbreviation: CI, confidence interval; GFR, glomerular filtration rate; HR, hazard ratio; PCI, percutaneous coronary intervention.

© 2023 Kwon W et al. *JAMA Network Open.*

eReferences

1. Witzenbichler B, Maehara A, Weisz G, et al. Relationship between intravascular ultrasound guidance and clinical outcomes after drug-eluting stents: the assessment of dual antiplatelet therapy with drug-eluting stents (ADAPT-DES) study. *Circulation* 2014; **129**(4): 463-70.
2. Chieffo A, Latib A, Caussin C, et al. A prospective, randomized trial of intravascular-ultrasound guided compared to angiography guided stent implantation in complex coronary lesions: the AVIO trial. *Am Heart J* 2013; **165**(1): 65-72.
3. Jakabcin J, Spacek R, Bystron M, et al. Long-term health outcome and mortality evaluation after invasive coronary treatment using drug eluting stents with or without the IVUS guidance. Randomized control trial. HOME DES IVUS. *Catheter Cardiovasc Interv* 2010; **75**(4): 578-83.
4. Kim JS, Kang TS, Mintz GS, et al. Randomized comparison of clinical outcomes between intravascular ultrasound and angiography-guided drug-eluting stent implantation for long coronary artery stenoses. *JACC Cardiovasc Interv* 2013; **6**(4): 369-76.
5. Kim BK, Shin DH, Hong MK, et al. Clinical Impact of Intravascular Ultrasound-Guided Chronic Total Occlusion Intervention With Zotarolimus-Eluting Versus Biolimus-Eluting Stent Implantation: Randomized Study. *Circulation Cardiovascular interventions* 2015; **8**(7): e002592.
6. Hong SJ, Kim BK, Shin DH, et al. Effect of Intravascular Ultrasound-Guided vs Angiography-Guided Everolimus-Eluting Stent Implantation: The IVUS-XPL Randomized Clinical Trial. *JAMA* 2015; **314**(20): 2155-63.
7. Hong SJ, Mintz GS, Ahn CM, et al. Effect of Intravascular Ultrasound-Guided Drug-Eluting Stent Implantation: 5-Year Follow-Up of the IVUS-XPL Randomized Trial. *JACC Cardiovasc Interv* 2020; **13**(1): 62-71.
8. Gao XF, Ge Z, Kong XQ, et al. 3-Year Outcomes of the ULTIMATE Trial Comparing Intravascular Ultrasound Versus Angiography-Guided Drug-Eluting Stent Implantation. *JACC Cardiovasc Interv* 2021; **14**(3): 247-57.
9. Shin DH, Kang HJ, Jang JS, et al. The Current Status of Percutaneous Coronary Intervention in Korea: Based on Year 2014 & 2016 Cohort of Korean Percutaneous Coronary Intervention (K-PCI) Registry. *Korean circulation journal* 2019; **49**(12): 1136-51.
10. Garcia-Garcia HM, McFadden EP, Farb A, et al. Standardized End Point Definitions for Coronary Intervention Trials: The Academic Research Consortium-2 Consensus Document. *Circulation* 2018; **137**(24): 2635-50.
11. Thygesen K, Alpert JS, Jaffe AS, et al. Third universal definition of myocardial infarction. *Circulation* 2012; **126**(16): 2020-35.
12. Moussa ID, Klein LW, Shah B, et al. Consideration of a new definition of clinically relevant myocardial infarction after coronary revascularization: an expert consensus document from the Society for Cardiovascular Angiography and Interventions (SCAI). *J Am Coll Cardiol* 2013; **62**(17): 1563-70.
13. Bangalore S, Toklu B, Amoroso N, et al. Bare metal stents, durable polymer drug eluting stents, and biodegradable polymer drug eluting stents for coronary artery disease: mixed treatment comparison meta-analysis. *BMJ* 2013; **347**: f6625.
14. Raber L, Mintz GS, Koskinas KC, et al. Clinical use of intracoronary imaging. Part 1: guidance and optimization of coronary interventions. An expert consensus document of the European Association of Percutaneous Cardiovascular Interventions. *Eur Heart J* 2018; **39**(35): 3281-300.
15. Levine GN, Bates ER, Bittl JA, et al. 2016 ACC/AHA Guideline Focused Update on

© 2023 Kwon W et al. *JAMA Network Open.*

Duration of Dual Antiplatelet Therapy in Patients With Coronary Artery Disease: A Report of the American College of Cardiology/American Heart Association Task Force on Clinical Practice Guidelines: An Update of the 2011 ACCF/AHA/SCAI Guideline for Percutaneous Coronary Intervention, 2011 ACCF/AHA Guideline for Coronary Artery Bypass Graft Surgery, 2012 ACC/AHA/ACP/AATS/PCNA/SCAI/STS Guideline for the Diagnosis and Management of Patients With Stable Ischemic Heart Disease, 2013 ACCF/AHA Guideline for the Management of ST-Elevation Myocardial Infarction, 2014 AHA/ACC Guideline for the Management of Patients With Non–ST-Elevation Acute Coronary Syndromes, and 2014 ACC/AHA Guideline on Perioperative Cardiovascular Evaluation and Management of Patients Undergoing Noncardiac Surgery. *Circulation* 2016; **134**(10): e123-e55.

16. Valgimigli M, Bueno H, Byrne RA, et al. 2017 ESC focused update on dual antiplatelet therapy in coronary artery disease developed in collaboration with EACTS: The Task Force for dual antiplatelet therapy in coronary artery disease of the European Society of Cardiology (ESC) and of the European Association for Cardio-Thoracic Surgery (EACTS). *European Heart Journal* 2018; **39**(3): 213-60.

17. Lawton JS, Tamis-Holland JE, Bangalore S, et al. 2021 ACC/AHA/SCAI Guideline for Coronary Artery Revascularization: A Report of the American College of Cardiology/American Heart Association Joint Committee on Clinical Practice Guidelines. *Circulation* 2022; **145**(3): e18-e114.

18. Neumann FJ, Sousa-Uva M, Ahlsson A, et al. 2018 ESC/EACTS Guidelines on myocardial revascularization. *Eur Heart J* 2019; **40**(2): 87-165.

© 2023 Kwon W et al. *JAMA Network Open.*
