# Supplementary material for: Intravascular Imaging in Patients With Complex Coronary Lesions and Chronic Kidney Disease
Source: JAMA Netw Open. 2023 Nov 29;6(11):e2345554. doi: 10.1001/jamanetworkopen.2023.45554 (PMC10687657; doi:10.1001/jamanetworkopen.2023.45554)
Supplement: Supplement 2. — Nonauthor Collaborators [file jamanetwopen-e2345554-s002.pdf]

Supplemental Online Content: Nonauthor Collaborators

*Indicates required information. Only first name, last name, and suffix will appear in PubMed.

*Group Name(s): The RENOVATE COMPLEX-PCI Investigators							
***First Name and Middle Initial(s)**	***Last Name**	***Suffix (eg, Jr, III)**	Academic Degrees	Institution	Location (city, state/province, country)	Role or Contribution, eg, chair, principal investigator	Group (if more than 1 Group listed in the byline) and/or Subgroup (eg, Steering Committee)
Hyun-Jong	Lee		MD, PhD	Sejong General Hospital	Bucheon, Korea		Clinical Event Adjudication Committee
Dong R	Ryu		MD, PhD	Kangwon National University Hospital, Kangwon National University School of Medicine	Chuncheon, Korea		Clinical Event Adjudication Committee
Kyu T	Park		MD, PhD	Chuncheon Sacred Heart Hospital, Hallym University College of Medicine	Chuncheon, Korea		Clinical Event Adjudication Committee
Kiyuk	Chang		MD, PhD	Seoul St. Mary's Hospital, The Catholic University of Korea	Seoul, Korea		Data Safety Monitoring Board
Seonwoo	Kim		PhD	Academic Research Service Headquarter, LSK Global PS	Seoul, Korea		Data Safety Monitoring Board
Dong-Yeon	Kim		MD, PhD	Seoul Medical Center	Seoul, Korea		Data Safety Monitoring Board
Suyoun	Shin		RN	Heart Vascular Stroke Institute, Samsung Medical Center, Sungkyunkwan University School of Medicine	Seoul, Korea		Data Coordination and Management
Jinshil	Kim		RN	Heart Vascular Stroke Institute, Samsung Medical Center, Sungkyunkwan University School of Medicine	Seoul, Korea		Data Coordination and Management
Jaeyoung	Park		RN	Heart Vascular Stroke Institute, Samsung Medical Center, Sungkyunkwan University School of Medicine	Seoul, Korea		Data Coordination and Management
Seunghyun	Lee		RN	Heart Vascular Stroke Institute, Samsung Medical Center, Sungkyunkwan University School of Medicine	Seoul, Korea		Data Coordination and Management

Supplemental Online Content: Nonauthor Collaborators

*Indicates required information. Only first name, last name, and suffix will appear in PubMed.

***First Name and Middle Initial(s)**	***Last Name**	***Suffix (eg, Jr, III)**	Academic Degrees	Institution	Location (city, state/province, country)	Role or Contribution, eg, chair, principal investigator	Group (if more than 1 Group listed in the byline) and/or Subgroup (eg, Steering Committee)
Euna	Kim		RN	Heart Vascular Stroke Institute, Samsung Medical Center, Sungkyunkwan University School of Medicine	Seoul, Korea		Data Coordination and Management
Hyein	Kang		RT	Heart Vascular Stroke Institute, Samsung Medical Center, Sungkyunkwan University School of Medicine	Seoul, Korea		Data Coordination and Management, Angiography Core Laboratory, Intravascular Imaging Core Laboratory
Su J	Hwang			Heart Vascular Stroke Institute, Samsung Medical Center, Sungkyunkwan University School of Medicine	Seoul, Korea		Data Coordination and Management
Yeonhui	Lee			Heart Vascular Stroke Institute, Samsung Medical Center, Sungkyunkwan University School of Medicine	Seoul, Korea		Data Coordination and Management
Hyun S	Joh		MD	Seoul National University Boramae Medical Center, Seoul National University College of Medicine	Seoul, Korea		Angiography Core Laboratory
Se Y	Im		RT	Heart Vascular Stroke Institute, Samsung Medical Center, Sungkyunkwan University School of Medicine	Seoul, Korea		Intravascular Imaging Core Laboratory
