# Supplementary material for: Intravascular Imaging in Patients With Complex Coronary Lesions and Chronic Kidney Disease
Source: JAMA Netw Open. 2023 Nov 29;6(11):e2345554. doi: 10.1001/jamanetworkopen.2023.45554 (PMC10687657; doi:10.1001/jamanetworkopen.2023.45554)
Supplement: Supplement 3. — Data Sharing Statement [file jamanetwopen-e2345554-s003.pdf]

Data Sharing Statement

Kwon. Intravascular Imaging in Patients With Complex Coronary Lesions and Chronic Kidney Disease. *JAMA Netw Open*. Published November 29, 2023.
doi:10.1001/jamanetworkopen.2023.45554

Data
Data available: No
